# Fluoroquinolone‐Associated Psychiatric and Ocular Adverse Events: A Disproportionality Analysis Using Real‐World Data From FAERS (2011–2024)

**DOI:** 10.1002/prp2.70206

**Published:** 2025-12-17

**Authors:** Hau‐Tak Chau, Ngan Pan Bennett Au

**Affiliations:** ^1^ Department of Medicine, School of Clinical Medicine The University of Hong Kong Hong Kong Hong Kong SAR; ^2^ Department of Comparative Biomedical Sciences, School of Veterinary Medicine University of Surrey Guildford UK

**Keywords:** antibiotics, eye disorders, FDA adverse event reporting system (FAERS), fluoroquinolone, pharmacovigilance, psychiatric disorders

## Abstract

Fluoroquinolones (FQs) are widely prescribed antibiotics; however, increasing concerns have arisen over their potential to cause disabling and irreversible adverse effects. Using real‐world data from the FDA Adverse Event Reporting System (FAERS) (2011–2024), we performed disproportionality analyses on psychiatric and eye‐related adverse events (AEs) using reporting odds ratios (RORs). Of 44 895 FQ‐associated adverse event reports, 8518 cases exhibited psychiatric AEs whilst 4367 cases displayed eye‐related AEs. Among six FDA‐approved FQs, three (ciprofloxacin/levofloxacin/moxifloxacin) showed safety signals for psychiatric disorders and four (ciprofloxacin/levofloxacin/moxifloxacin/ofloxacin) for eye‐related disorders. Eight psychiatric and nine eye‐related AEs were commonly reported across multiple FQs. Importantly, moxifloxacin exhibited unique safety signals for ocular‐related toxicity, including iris transillumination defect [ROR: 6604.89 (4736.76–9209.81)], iris hypopigmentation [1887.66 (1175.44–3031.42)], and pigment dispersion syndrome [2360.62 (1432.71–3889.51)], raising concern for potential progression to pigmentary glaucoma—findings not prominently featured in current safety warnings. Additionally, systematic characterization of suicide cases revealed striking male predominance despite females comprising the majority of psychiatric AEs, identifying middle‐aged males as a high‐risk demographic. These findings support the need for enhanced pharmacovigilance, particularly regarding moxifloxacin's ocular toxicity and FQs' suicide vulnerability in specific patient subgroups. When FQ prescription becomes unavoidable, robust safety monitoring should be implemented to detect early neuropsychiatric and ocular complications.

## Graphical abstract

This pharmacovigilance study identifies a wide range of rapid‐onset neuropsychiatric and ocular adverse effects linked to fluoroquinolones (FQ). Disproportionality analysis revealed a disproportionate reporting of suicidal ideation and visual impairment following FQ treatment, emphasizing the urgent need for cautious prescribing and strengthened safety monitoring of this widely used antibiotic class.

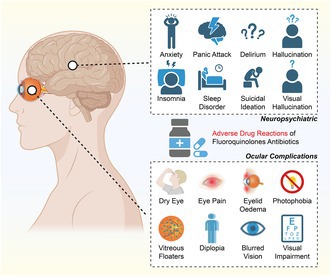

## 1 Introduction

Fluoroquinolones (FQs) constitute a class of broad‐spectrum antibiotics for treating bacterial infections of the urinary, respiratory, and gastrointestinal tracts, as well as ocular infections caused predominantly by Gram‐negative bacteria, with certain FQs demonstrating efficacy against Gram‐positive bacteria [1, 2]. Their mechanism of action involves inhibition of two essential bacterial enzymes required for DNA replication: DNA gyrase and topoisomerase IV [3]. Owing to their efficacy and broad‐spectrum coverage, FQs have become among the most frequently prescribed antibiotics in both human and veterinary medicine [4, 5]. In the United States alone, more than 10 million prescriptions are documented annually [6], with four FQs (ciprofloxacin, levofloxacin, moxifloxacin, and ofloxacin) featuring in the top 300 most commonly used medications [7]. Across Europe, FQs ranked as the fifth most commonly used antibiotic class, accounting for 9.47% of overall antibiotic usage [8]. Despite their extensive clinical applications, increasing concerns have arisen over serious adverse drug reactions linked to FQ treatment, including tendinopathy and tendon rupture, aortic aneurysm and dissection, peripheral neuropathy, cardiac arrhythmias, central nervous system (CNS) effects, and retinal detachment [9]. Whilst FQ usage in Europe has plateaued in recent years [10], its consumption has expanded rapidly across North Africa and the Middle East over the past decade [11]. Given their global prevalence, understanding the full spectrum of FQ‐associated adverse effects has become increasingly critical.

In response to accumulating safety concerns, the U.S. Food and Drug Administration (FDA) issued its first boxed warning for FQs in 2008, alerting healthcare professionals and patients to the increased risk of tendinitis and tendon rupture [12]. This warning was strengthened in 2016, highlighting the potential disabling and irreversible side effects involving tendons, muscles, joints, peripheral nerves, and the CNS associated with FQ treatment. In 2017–2018, the FDA further cautioned against risks of retinal detachment and mental health conditions, ultimately advising healthcare professionals to reserve FQ use for patients with bacterial infections “who have no alternative treatment options” [13]. Other major regulatory authorities, including the European Medicines Agency (EMA), the Medicines and Healthcare products Regulatory Agency (MHRA), and the Therapeutic Goods Administration (TGA), subsequently introduced similar restrictions in 2019, 2024, and 2025, respectively. While the neuropsychiatric effects of FQs are increasingly recognized [14, 15], recent pharmacovigilance studies based on real‐world data have yet to provide a comprehensive analysis encompassing all six FDA‐approved FQs (i.e., ciprofloxacin, levofloxacin, moxifloxacin, ofloxacin, gemifloxacin, and delafloxacin) [16, 17]. For FQ‐associated ocular disorders, only one pharmacovigilance analysis has been conducted using real‐world data, which exclusively focused on children and adolescents under 18 years of age [18]. Consequently, a systematic investigation into both psychiatric and eye‐related adverse events (AEs) across all FDA‐approved FQs is warranted to uncover novel safety signals and support risk management of this potent antibiotic class. Disproportionality analysis using real‐world data from databases such as the FDA Adverse Events Reporting System (FAERS) enables systematic, large‐scale quantification of safety signals that complements conventional clinical studies, enabling the detection of rare, serious, or previously under‐recognized AEs that are not captured in registration trials.

Here, we conducted a comprehensive pharmacovigilance analysis using FAERS Public Dashboard, covering the period from 2011 to 2024. We identified 44 895 adverse event reports (AERs) associated with all six FDA‐approved FQs. Among these reports, 8518 cases (18.97% of total FQ‐associated AERs) presented with psychiatric AEs, whilst 4367 cases (9.73% of total FQ‐associated AERs) exhibited eye‐related AEs. Using the complete FAERS dataset from 2011 to 2024 as the comparator, our disproportionality analyses revealed eight psychiatric and nine eye‐related AEs that emerged as common positive safety signals reported by at least four FQs. Additionally, we uncovered drug‐specific AEs, with moxifloxacin showing disproportionate reporting of structural alterations in the anterior segment of the eyes, potentially relevant to glaucoma. Importantly, suicide was disproportionately reported among mortality cases of FQ patients experiencing psychiatric AEs. Taken together, our findings provide important real‐world evidence on neuropsychiatric and ocular complications associated with FQs, offering valuable insights to inform clinical decision‐making and pharmacovigilance practices.

## 2 Methods

### 2.1 Source of Data: FDA Adverse Event Reporting System (FAERS)

Pharmacovigilance analyses of psychiatric and eye‐related adverse events (AEs) associated with fluoroquinolone (FQ) treatment were conducted using the FDA Adverse Event Reporting System (FAERS) Public Dashboard. This interactive web‐based resource contains over 30 million self‐reported adverse event reports (AERs) submitted globally by consumers, healthcare professionals, and pharmaceutical companies from 1968 to 2024. Each AER documented patient demographics, medication usage, suspected drugs triggering AEs, clinical indications, outcomes, and reporting sources. We extracted all AERs involving six FDA‐approved FQs (ciprofloxacin, levofloxacin, moxifloxacin, ofloxacin, gemifloxacin, and delafloxacin) from 2011 until 31 December 2024 (the latest data available during data extraction on 21 February 2025). Adverse drug reactions were coded according to Preferred Terms (PTs) from the Medical Dictionary for Regulatory Activities (MedDRA) version 27.1. To identify psychiatric and eye‐related AERs linked to FQ treatment, we included all PTs categorized under the System Organ Classes (SOCs) of “psychiatric disorders” (551 PTs in total) and “eye disorders” (653 PTs) as their primary classification.

### 2.2 Data Processing

All AERs extracted from the FAERS Public Dashboard (2011–2024) were first subjected to deduplication by identifying reports with identical patient demographics (e.g., age, sex, country), medical information (e.g., drug indications, clinical indications), reporting details (initial FDA received date), and adverse reactions, in accordance with World Health Organization (WHO) Uppsala Monitoring Centre guidelines [19]. The deduplicated dataset was further filtered to exclude cases with concomitant medications known to potentially induce psychiatric adverse effects (i.e., any medications classified under Anatomical Therapeutic Chemical codes N05 Psycholeptics, N06 Psychoanaleptics, and N07B Drugs Used in Addictive Disorders). The resulting final dataset comprised 44 895 cases with the six FDA‐approved FQs as suspected drugs for subsequent analysis (Figure S1).

### 2.3 Descriptive Analysis

Following deduplication and additional filtering, we conducted descriptive analysis of clinical characteristics for all psychiatric and eye‐related AERs associated with FQ treatment. This included sex, age, age group, reporting country, reporter type, and clinical outcomes. Life‐threatening events, hospitalization, disability, and death were considered as serious outcomes. Between‐group differences in categorical variables were assessed using Chi‐square test or Fisher's exact test where appropriate [20].

### 2.4 Disproportionality Analysis

Pharmacovigilance disproportionality analyses were performed using reporting odds ratio (ROR), a well‐established and most widely used approach to identify potential novel safety signals associated with FQs [21]. The complete FAERS dataset from 2011 to 2024 served as the comparator [20].

Prior to analysis, drug adverse reaction contingency tables were constructed comprising: (a) psychiatric or eye‐related AEs following FQ treatment; (b) non‐psychiatric or non‐eye‐related AEs following FQ treatment; (c) psychiatric or eye‐related AEs without FQ treatment; and (d) non‐psychiatric or non‐eye‐related AEs without FQ treatment. The ROR and 95% confidence interval (CI) for each PT were calculated using the faers R package [22]. A positive signal was defined as an ROR exceeding 1 with the lower limit of the 95% CI also exceeding 1, with a minimum of 3 reported cases within the study period [20].

### 2.5 Time‐to‐Onset (TTO) Analysis

In the FAERS database, time‐to‐onset was defined as the interval between the start date of drug administration (i.e., START_DT) and the date when the AEs first occurred (i.e., EVENT_DT). Cumulative distribution curves were used to illustrate the time‐to‐onset patterns of psychiatric and eye‐related AEs following FQ treatment across various subgroups (e.g., FQ monotherapy versus combination therapy, sex and age group) based on FAERS data [23].

### 2.6 Statistical Analysis

Between‐group differences in categorical variables were evaluated using the Chi‐square test or Fisher's exact test where appropriate [20]. For TTO analysis, cumulative distribution curves were plotted using the survival [24] and survminer R packages [25]. Between‐group comparisons were conducted using the Wilcoxon rank‐sum test, while pairwise comparisons across multiple groups were assessed using the Kruskal–Wallis test. *p*‐values were adjusted using the Benjamini–Hochberg correction to control for false discovery rate. Odds ratios (ORs) for psychiatric or eye‐related AEs associated with FQ treatment were calculated using univariate logistic regression analysis with the MASS [26] and broom R packages [27]. Before regression analysis, missing age and sex data were imputed using the k‐nearest neighbors (kNN) algorithm [23] using the VIM R package [28]. *p*‐values were adjusted for individual FQ use (or combination therapy), age, sex and clinical outcomes using the “*p*‐adjust” function in RStudio. All statistical analyses were conducted using R software (version 4.4.3), and data were visualized using the ggplot2 R package [29]. A two‐sided *p*‐value less than 0.05 was considered statistically significant for all analyses, with the Benjamini–Hochberg correction applied to adjust for multiple comparisons.

### 2.7 Ethical Statement

This study analyzed data exclusively from the FDA Adverse Event Reporting System (FAERS), a publicly available database containing only anonymized and de‐identified patient records. Therefore, review and approval from the institutional ethical committee and individual informed consent were exempted from this study.

## 3 Results

### 3.1 Fluoroquinolone (FQ)‐Associated Adverse Events (AEs) in the FAERS Database, 2011–2024

The present study examined psychiatric and eye‐related adverse events (AEs) in patients treated with six FDA‐approved FQs (ciprofloxacin, levofloxacin, moxifloxacin, ofloxacin, gemifloxacin, and delafloxacin) using the publicly available FAERS database spanning 2011–2024 (Figure S1). Following World Health Organization (WHO) Uppsala Monitoring Centre guidelines [19], we established exclusion criteria to eliminate duplicated cases and those involving concomitant medications known to induce psychiatric symptoms, including antipsychotics, anxiolytics, hypnotics, sedatives, antidepressants, and psychostimulants (see Methods for detailed exclusion criteria). This identified a total of 44 895 adverse event reports (AERs) over the 14‐year period for descriptive analysis.

**FIGURE 1:**
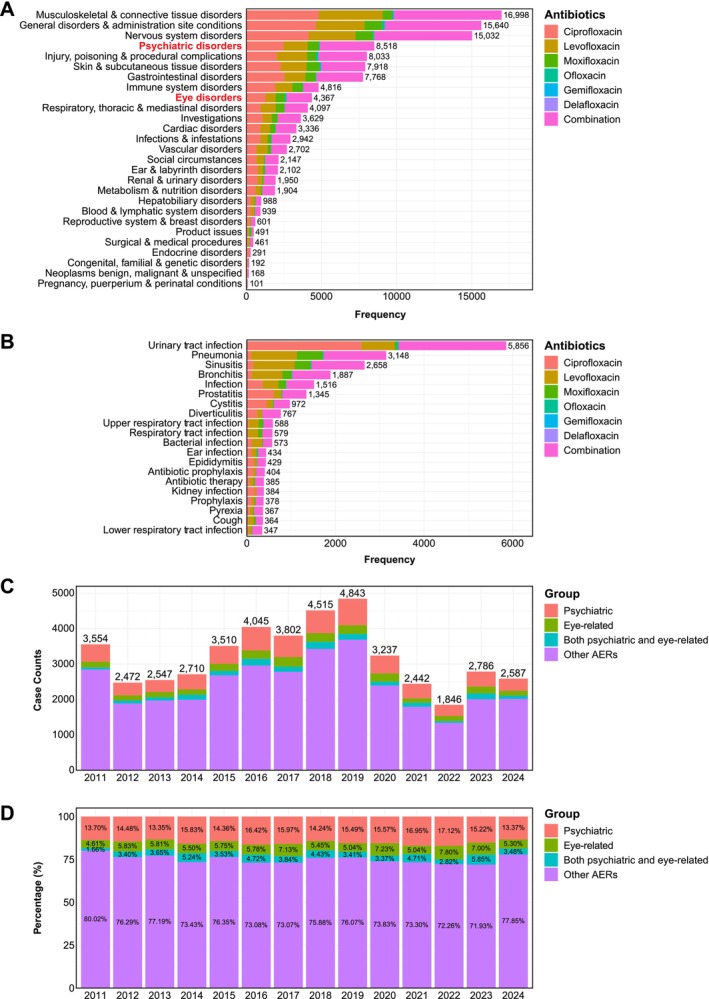
Overview of psychiatric and eye‐related adverse events (AEs) associated with fluoroquinolone (FQ) treatment, based on adverse event reports (AERs) from the FDA Adverse Event Reporting System (FAERS), 2011–2024. (A) Each Preferred Term (PT) was mapped to its primary System Organ Class (SOC), and the frequency of PTs under each SOC was ranked for individual FQs analyzed (ciprofloxacin, levofloxacin, moxifloxacin, ofloxacin, gemifloxacin, delafloxacin and combinations). Among all SOCs, psychiatric and eye disorders ranked 4th and 9th, respectively. (B) Clinical indications for FQ usage were ranked, revealing that FQs were predominantly prescribed for bacterial infections. (C) Stacked histogram showing the annual cases of psychiatric, eye‐related, combined psychiatric and eye‐related, and other AERs from 2011 to 2024. (D) Stacked histogram illustrating the proportional distribution of psychiatric, eye‐related, combined psychiatric and eye‐related, and other AERs throughout the study period.

Within FAERS, all AEs were classified according to MedDRA Preferred Terms (PTs). We initially mapped each PT from all AERs to its corresponding primary system organ class (SOC), revealing musculoskeletal and connective tissue disorders as the predominant AEs, constituting 37.9% (16 998/44895) of all FQ‐associated AERs (Figure 1A). This finding aligns with previous findings, given that tendinitis and tendon rupture represent the most common adverse side effects that prompted the first boxed warning for FQs issued by the FDA in 2008 [13]. Notably, psychiatric and eye disorders ranked as the 4th and 9th most prevalent AEs, accounting for 18.97% (8518/44 895) and 9.73% (4367/44 895) of all FQ‐associated AERs, respectively (Figure 1A). As anticipated, the primary clinical indications for FQ treatment in the reported AERs were urinary tract infections [13.04% (5856/44 895)], followed by pneumonia [7.01% (3148/44 895)], sinusitis [5.92% (2658/44 895)], and bronchitis [4.20% (1887/44 895)] (Figure 1B). Notably, a considerable proportion [0.81% (364/44 895)] of patients received FQs for cough, despite their potential associations with upper respiratory tract infections (Figure 1B). Our data otherwise did not indicate substantial off‐label uses of FQs.

Despite multiple boxed warnings issued by the FDA between 2008 and 2023, we observed a consistently high number of FQ‐associated AERs reported globally throughout the study period, exceeding 2000 cases recorded annually (except in 2022) (Table S1). Annual reporting showed a median of 458 [95% CI (316–664)] and 179.5 (123–246) FQ‐associated AERs that showed psychiatric and eye‐related AEs, respectively. Interestingly, a subset of reports [median: 119.5 (51–191)] demonstrated concurrent psychiatric and eye‐related AEs (Figure 1C). The proportion of AERs featuring psychiatric [median: 18.92% (15.85%–21.90%)] and eye‐related AEs [9.81% (6.98%–12.24%)] remained relatively stable throughout the study period (Figure 1D).

### 3.2 Descriptive Analysis of FQ‐Associated Psychiatric and Eye‐Related AERs

**FIGURE 2:**
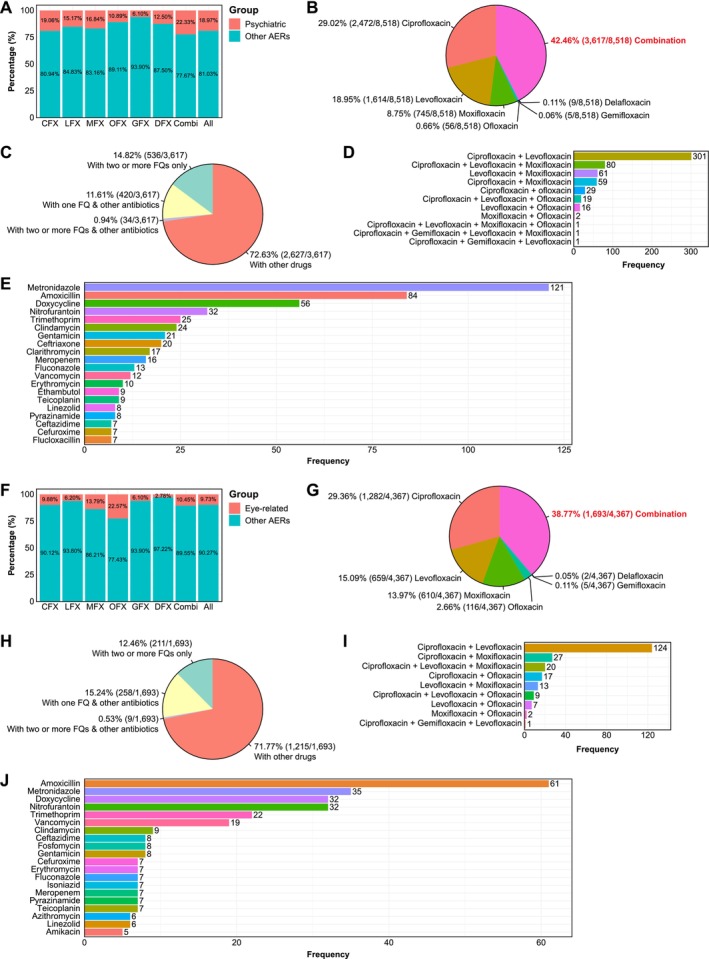
Characteristics of FQ‐associated psychiatric and eye‐related adverse events (AEs). (A) Overall, approximately 20% of total FQ‐associated AERs presented with psychiatric AEs. (B) Among psychiatric AERs, ciprofloxacin was the most frequently implicated FQ, with combination treatment representing a substantial proportion of the total cases. (C) Of the FQ‐associated psychiatric AERs involving combination therapy, most patients received FQs with other medications, while a considerable proportion received either multiple FQs or at least one FQ with other antibiotics. (D) The most frequent combination FQ treatment comprised ciprofloxacin and levofloxacin, with or without moxifloxacin. (E) Metronidazole emerged as the most commonly co‐administered antibiotic with FQs. (F) Eye‐related AEs accounted for approximately 10% of total FQ‐associated AERs, except for ofloxacin, which accounted for 22.57% of its total AERs. (G) Ciprofloxacin was the most frequently reported FQ among eye‐related AERs, with a substantial number involving combination therapies. (H) As with psychiatric AEs, most FQ‐associated eye‐related AERs with combination therapy involved at least one FQ with other medications, while a sizeable proportion involved multiple FQs or FQ(s) with other antibiotics. (I) Among reports involving two or more FQs, the most common combination was ciprofloxacin and levofloxacin. (J) Amoxicillin was the most frequently co‐administered antibiotic alongside FQ treatment in eye‐related AERs.

Of all FQ‐associated AERs, a total of 8518 cases (18.97%) presented with psychiatric AEs. The proportion of psychiatric AEs varied among different FQs: ciprofloxacin accounted for the highest frequency [19.06% (2472/12 970)], followed by moxifloxacin [16.84% (745/4425)] and levofloxacin [15.17% (1614/10 636)]. Gemifloxacin exhibited the lowest frequency of reported psychiatric AEs [6.10% (5/82)]. Notably, the proportion increased to 22.33% (3617/16 196) when combination therapy (either multiple FQs or FQs with other antibiotics or drugs) was reported (Figure 2A). Female patients constituted the majority of the cases [55.80% (4753/8518)] compared to males [38.20% (3254/8518)] (Table 1). This gender distribution aligns with our descriptive analysis of overall FQ‐associated AERs, where females accounted for 53.50% (24 018/44 895) of total AERs versus 37.25% (16 722/44 895) for males (Table S2). Detailed descriptive analysis of FQ‐associated AERs with psychiatric AEs is presented in Table 1.

**TABLE 1:** Demographic characteristics of fluoroquinolone (FQ)‐associated adverse event reports (AERs) with psychiatric adverse events (AEs).

	Ciprofloxacin (*N* = 2472)	Levofloxacin (*N* = 1614)	Moxifloxacin (*N* = 745)	Ofloxacin (*N* = 56)	Gemifloxacin (*N* = 5)	Delafloxacin (*N* = 9)	Combination (*N* = 3617)	Total (*N* = 8518)	*p*
*Sex*									9.01E−20
Male	1028	529	327	22	1	3	1344	3254	
	(41.59%)	(32.78%)	(43.89%)	(39.29%)	(20.00%)	(33.33%)	(37.16%)	(38.20%)	
Female	1314	910	396	32	4	5	2092	4753	
	(53.16%)	(56.38%)	(53.15%)	(57.14%)	(80.00%)	(55.56%)	(57.84%)	(55.80%)	
Not specified	130	175	22	2	0	1	181	511	
	(5.26%)	(10.84%)	(2.95%)	(3.57%)	(0.00%)	(11.11%)	(5.00%)	(6.00%)	
*Age*									9.67E−56
Median	43	52	64	41.5	49	61	54	51	
(Min–Max)	(32–55)	(40–65)	(44–81)	(28.50–54.75)	(49.5–62.5)	(55–67)	(41–67)	(37–66)	
*Age group*									5.44E−113
0–17	28	15	3	8	0	0	38	92	
	(1.13%)	(0.93%)	(0.40%)	(14.29%)	(0.00%)	(0.00%)	(1.05%)	(1.08%)	
18–34	589	196	67	13	1	0	487	1353	
	(23.83%)	(12.14%)	(8.99%)	(23.21%)	(20.00%)	(0.00%)	(13.46%)	(15.88%)	
35–64	1116	722	243	19	2	1	1642	3745	
	(45.15%)	(44.73%)	(32.62%)	(33.93%)	(40.00%)	(11.11%)	(45.40%)	(43.97%)	
65+	290	332	310	10	1	1	935	1879	
	(11.73%)	(20.57%)	(41.61%)	(17.86%)	(20.00%)	(11.11%)	(25.85%)	(22.06%)	
Not specified	449	349	122	6	1	7	515	1449	
	(18.16%)	(21.62%)	(16.38%)	(10.71%)	(20.00%)	(77.78%)	(14.24%)	(17.01%)	
*Reported country or continent*									*p* < 0.0001
United States	565	589	204	4	2	7	1360	2731	
	(22.86%)	(36.49%)	(27.38%)	(7.14%)	(40.00%)	(77.78%)	(37.60%)	(32.06%)	
Great Britain	507	55	2	17	0	0	660	1241	
	(20.51%)	(3.41%)	(0.27%)	(30.36%)	(0.00%)	(0.00%)	(18.25%)	(14.57%)	
Europe	737	424	92	23	2	2	648	1928	
	(29.81%)	(26.27%)	(12.35%)	(41.07%)	(40.00%)	(22.22%)	(17.92%)	(22.63%)	
Asia	19	37	311	6	0	0	110	483	
	(0.77%)	(2.29%)	(41.74%)	(10.71%)	(0.00%)	(0.00%)	(3.04%)	(5.67%)	
North America	130	31	25	0	0	0	158	344	
	(5.26%)	(1.92%)	(3.36%)	(0.00%)	(0.00%)	(0.00%)	(4.37%)	(4.04%)	
South America	7	10	6	0	0	0	15	38	
	(0.28%)	(0.62%)	(0.81%)	(0.00%)	(0.00%)	(0.00%)	(0.41%)	(0.45%)	
Africa	4	3	2	0	0	0	8	17	
	(0.16%)	(0.19%)	(0.27%)	(0.00%)	(0.00%)	(0.00%)	(0.22%)	(0.20%)	
Middle East	7	1	1	0	0	0	15	24	
	(0.28%)	(0.06%)	(0.13%)	(0.00%)	(0.00%)	(0.00%)	(0.41%)	(0.28%)	
Australia or New Zealand	12	0	2	0	0	0	3	17	
	(0.49%)	(0.00%)	(0.27%)	(0.00%)	(0.00%)	(0.00%)	(0.08%)	(0.20%)	
Not specified	484	464	100	6	1	0	640	1695	
	(19.58%)	(28.75%)	(13.42%)	(10.71%)	(20.00%)	(0.00%)	(17.69%)	(19.90%)	
*Reporter type*									1.82E−112
Consumer	1880	993	276	39	3	2	2605	5798	
	(76.05%)	(61.52%)	(37.05%)	(69.64%)	(60.00%)	(22.22%)	(72.02%)	(68.07%)	
Healthcare professional	485	532	448	17	1	7	939	2429	
	(19.62%)	(32.96%)	(60.13%)	(30.36%)	(20.00%)	(77.78%)	(25.96%)	(28.52%)	
Not specified	107	89	21	0	1	0	73	291	
	(4.33%)	(5.51%)	(2.82%)	(0.00%)	(20.00%)	(0.00%)	(2.02%)	(3.42%)	
*Outcomes*									7.92E−46
Died	38	21	9	2	0	0	73	143	
	(1.54%)	(1.30%)	(1.21%)	(3.57%)	(0.00%)	(0.00%)	(2.02%)	(1.68%)	
Life‐threatening	142	123	48	3	0	0	233	549	
	(5.74%)	(7.62%)	(6.44%)	(5.36%)	(0.00%)	(0.00%)	(6.44%)	(6.45%)	
Hospitalised	345	282	109	5	0	1	506	1248	
	(13.96%)	(17.47%)	(14.63%)	(8.93%)	(0.00%)	(11.11%)	(13.99%)	(14.65%)	
Disabled	743	377	60	12	0	0	1187	2379	
	(30.06%)	(23.36%)	(8.05%)	(21.43%)	(0.00%)	(0.00%)	(32.82%)	(27.93%)	
Other serious	1000	633	427	31	4	3	1308	3406	
	(40.45%)	(39.22%)	(57.32%)	(55.36%)	(80.00%)	(33.33%)	(36.16%)	(39.99%)	
Non‐serious	204	178	92	3	1	5	310	793	
	(8.25%)	(11.03%)	(12.35%)	(5.36%)	(20.00%)	(55.56%)	(8.57%)	(9.31%)	

Among all 8518 FQ‐associated AERs with psychiatric AEs, ciprofloxacin was most frequently reported [29.02% of total cases (2472/8518)], followed by levofloxacin [18.95% (1614/8518)] and moxifloxacin [8.75% (745/8518)]. The largest proportion [42.46% (3617/8518)] originated from patients receiving combination therapy (Figure 2B). Within this combination therapy group, the majority [72.63% (2627/3617)] involved at least one FQ combined with other medications (e.g., aspirin, levothyroxacin and acetaminophen were among the top of the list) (Figure 2C). A substantial proportion involved either multiple FQs [14.82% (536/3617)] (e.g., ciprofloxacin and levofloxacin) (Figure 2C,D) or at least one FQ with other antibiotics [12.55% (454/3617)], such as metronidazole and amoxicillin known for their capability of treating polymicrobial infections when combined with FQs (Figure 2E) [30].

We identified 4367 FQ‐associated AERs featuring eye‐related AEs, comprising 9.73% of all FQ‐associated AERs. Whilst most FQs were associated with eye‐related AEs in fewer than 10% of cases (ranging from 2.78% to 9.88%), ofloxacin [22.57% (116/514)] and moxifloxacin [13.79% (610/4425)] exhibited substantially higher proportions among other FQs (Figure 2F). Only 2 cases (2/72) from delafloxacin‐treated patients displayed eye‐related AEs, likely because this agent was only approved by the FDA in 2017 for acute bacterial skin infections and is currently undergoing phase 3 trials for pneumonia [31]. Table 2 presents a detailed descriptive analysis of FQ‐associated AERs with eye‐related AEs.

**TABLE 2:** Demographic characteristics of FQ‐associated AERs with eye‐related AEs.

	Ciprofloxacin (*N* = 1282)	Levofloxacin (*N* = 659)	Moxifloxacin (*N* = 610)	Ofloxacin (*N* = 116)	Gemifloxacin (*N* = 5)	Delafloxacin (*N* = 2)	Combination (*N* = 1693)	Total (*N* = 4367)	*p*
*Sex*									8.00E−19
Male	524	258	179	27	1	1	581	1571	
	(40.87%)	(39.15%)	(29.34%)	(23.28%)	(20.00%)	(50.00%)	(34.32%)	(35.97%)	
Female	684	313	351	81	4	1	1027	2461	
	(53.35%)	(47.50%)	(57.54%)	(69.83%)	(80.00%)	(50.00%)	(60.66%)	(56.35%)	
Not specified	74	88	80	8	0	0	85	335	
	(5.77%)	(13.35%)	(13.11%)	(6.90%)	(0.00%)	(0.00%)	(5.02%)	(7.67%)	
*Age*									6.80E−08
Median	45	49	53	47	44	Not Specified	50	49	
(Min–Max)	(34.00–57.75)	(38–60)	(40–64)	(29–63)	(36.50–49.25)		(36–64)	(36–62)	
*Age group*									1.59E−40
0–17	12	6	19	11	0	0	28	76	
	(0.94%)	(0.91%)	(3.11%)	(9.48%)	(0.00%)	(0.00%)	(1.65%)	(1.74%)	
18–34	267	92	62	12	1	0	302	736	
	(20.83%)	(13.96%)	(10.16%)	(10.34%)	(20.00%)	(0.00%)	(17.84%)	(16.85%)	
35–64	571	319	234	31	3	0	781	1939	
	(44.54%)	(48.41%)	(38.36%)	(26.72%)	(60.00%)	(0.00%)	(46.13%)	(44.40%)	
65+	164	88	101	15	0	0	362	730	
	(12.79%)	(13.35%)	(16.56%)	(12.93%)	(0.00%)	(0.00%)	(21.38%)	(16.72%)	
Not specified	268	154	194	47	1	2	220	886	
	(20.90%)	(23.37%)	(31.80%)	(40.52%)	(20.00%)	(100.00%)	(12.99%)	(20.29%)	
*Reported country or continent*									2.88E−94
United States	281	172	259	55	0	2	544	1313	
	(21.92%)	(26.10%)	(42.46%)	(47.41%)	(0.00%)	(100.00%)	(32.13%)	(30.07%)	
Great Britain	199	25	5	10	0	0	326	565	
	(15.52%)	(3.79%)	(0.82%)	(8.62%)	(0.00%)	(0.00%)	(19.26%)	(12.94%)	
Europe	420	259	139	29	3	0	337	1187	
	(32.76%)	(39.30%)	(22.79%)	(25.00%)	(60.00%)	(0.00%)	(19.91%)	(27.18%)	
Asia	30	19	67	2	0	0	68	186	
	(2.34%)	(2.88%)	(10.98%)	(1.72%)	(0.00%)	(0.00%)	(4.02%)	(4.26%)	
North America	92	8	28	0	0	0	89	217	
	(7.18%)	(1.21%)	(4.59%)	(0.00%)	(0.00%)	(0.00%)	(5.26%)	(4.97%)	
South America	7	5	32	0	0	0	11	55	
	(0.55%)	(0.76%)	(5.25%)	(0.00%)	(0.00%)	(0.00%)	(0.65%)	(1.26%)	
Africa	10	0	3	0	0	0	8	21	
	(0.78%)	(0.00%)	(0.49%)	(0.00%)	(0.00%)	(0.00%)	(0.47%)	(0.48%)	
Middle East	7	1	11	0	0	0	4	23	
	(0.55%)	(0.15%)	(1.80%)	(0.00%)	(0.00%)	(0.00%)	(0.24%)	(0.53%)	
Australia or New Zealand	4	0	1	0	0	0	5	10	
	(0.31%)	(0.00%)	(0.16%)	(0.00%)	(0.00%)	(0.00%)	(0.30%)	(0.23%)	
Not specified	232	170	65	20	2	0	301	790	
	(18.10%)	(25.80%)	(10.66%)	(17.24%)	(40.00%)	(0.00%)	(17.78%)	(18.09%)	
*Reporter type*									2.06E−36
Consumer	898	419	249	74	5	1	1110	2756	
	(70.05%)	(63.58%)	(40.82%)	(63.79%)	(100.00%)	(50.00%)	(65.56%)	(63.11%)	
Healthcare professional	334	210	348	34	0	1	550	1477	
	(26.05%)	(31.87%)	(57.05%)	(29.31%)	(0.00%)	(50.00%)	(32.49%)	(33.82%)	
Not specified	50	30	13	8	0	0	33	134	
	(3.90%)	(4.55%)	(2.13%)	(6.90%)	(0.00%)	(0.00%)	(1.95%)	(3.07%)	
*Outcomes*									5.45E−53
Died	6	7	3	0	0	0	21	37	
	(0.47%)	(1.06%)	(0.49%)	(0.00%)	(0.00%)	(0.00%)	(1.24%)	(0.85%)	
Life‐threatening	64	64	28	1	0	0	98	255	
	(4.99%)	(9.71%)	(4.59%)	(0.86%)	(0.00%)	(0.00%)	(5.79%)	(5.84%)	
Hospitalised	145	70	63	6	2	0	149	435	
	(11.31%)	(10.62%)	(10.33%)	(5.17%)	(40.00%)	(0.00%)	(8.80%)	(9.96%)	
Disabled	373	196	64	14	1	0	602	1250	
	(29.10%)	(29.74%)	(10.49%)	(12.07%)	(20.00%)	(0.00%)	(35.56%)	(28.62%)	
Other serious	551	271	323	43	2	1	669	1860	
	(42.98%)	(41.12%)	(52.95%)	(37.07%)	(40.00%)	(50.00%)	(39.52%)	(42.59%)	
Non‐serious	143	51	129	52	0	1	154	530	
	(11.15%)	(7.74%)	(21.15%)	(44.83%)	(0.00%)	(50.00%)	(9.10%)	(12.14%)	

Within all six FDA‐approved FQs, ciprofloxacin was most frequently implicated in eye‐related AEs [29.36% (1282/4367)], followed by levofloxacin [15.09% (659/4367)] and moxifloxacin [13.97% (610/4367)] (Figure 2G). Among the 1693 cases receiving combination therapy, 71.77% (1215/1693) involved FQs combined with other medications (e.g., aspirin, levothyroxine, and ibuprofen were among the top of the list) (Figure 2H). The remaining cases involved either multiple FQs [12.46% (211/1693)] (e.g., ciprofloxacin and levofloxacin) (Figure 2I), or at least one FQ with other antibiotics [15.77% (267/1693)], such as amoxicillin and metronidazole (Figure 2J).

### 3.3 Identification of FQ‐Associated Psychiatric and Eye‐Related AEs

In 2018, the US FDA reinforced safety warnings regarding mental health‐related side effects of FQs, including anxiety, hallucinations, delirium, memory impairment and attention disturbances [15]. Several studies also indicated that patients receiving FQ treatment posed an elevated risk of retinal detachment [32, 33], suggesting potential ocular toxicity. Our descriptive analyses revealed that psychiatric and eye‐related AEs accounted for 18.97% and 9.73% of total AERs associated with FQ treatment, respectively, underscoring the importance of a systematic evaluation of these potential risks.

**FIGURE 3:**
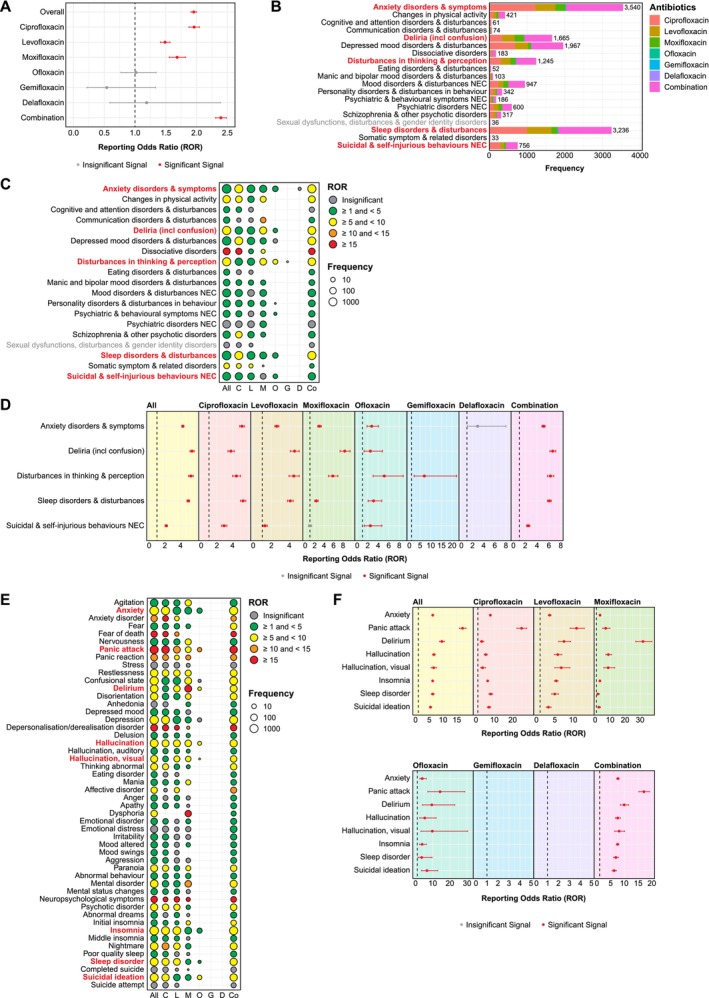
Disproportionality analysis reveals significant psychiatric safety signals associated with FQ treatment based on FAERS data. (A) Disproportionality analysis was conducted on six FDA‐approved FQs (ciprofloxacin, levofloxacin, moxifloxacin, ofloxacin, gemifloxacin, and delafloxacin) using reporting odds ratio (ROR). Among all FQs, ciprofloxacin, levofloxacin and moxifloxacin demonstrated significant safety signals associated with psychiatric AEs. (B) Each psychiatric PT was mapped to its primary High Level Group Term (HLGT). “Anxiety disorders and symptoms” and “sleep disorders and disturbances” emerged as the most frequent HLGTs following FQ treatment. (C) Heat map illustrating ROR values across all psychiatric HLGTs. Dots represented HLGTs with at least three reported cases in FAERS, with dot size proportional to the frequency for individual agents (C: ciprofloxacin; L: levofloxacin; M: moxifloxacin; O: ofloxacin; G: gemifloxacin; D: delafloxacin; Co: combination therapy). HLGT terms highlighted in red indicated common positive safety signals reported by at least four FQs. (D) Forest plots displaying ROR values (with 95% confidence intervals; CI) of common HLGT signals reported by at least four FQs. Red denoted significant positive signals whilst gray indicated insignificant signals. (E) Heat map showing ROR values for the 50 most common psychiatric PTs. Dot represented PTs with a minimum of three cases in FAERS, with dot sizes proportional to their frequency for each FQ. PT terms highlighted in red indicated common positive safety signals reported by at least four FQs. (F) Forest plots presenting ROR values (with 95% CI) for eight psychiatric PTs representing common safety signals reported by at least four FQs. Red denoted significant positive signals whilst gray indicated insignificant signals.

To comprehensively assess safety signals across different FQ antibiotics and their associations with psychiatric AEs, we first conducted disproportionality analyses for each FQ by calculating the reporting odds ratio (ROR), using the complete FAERS database (2011–2024) as a comparator. A signal was considered significant if the ROR exceeded 1 and the lower limit of its 95% confidence interval (CI) was also greater than 1 [20]. Overall, FQ treatment demonstrated disproportionate reporting of psychiatric AEs [ROR: 1.95 (1.91–2.00)]. Among the six FDA‐approved FQs, three demonstrated significant safety signals: ciprofloxacin [1.96 (1.88–2.05)], moxifloxacin [1.68 (1.56–1.82)], and levofloxacin [1.49 (1.41–1.57)]. Notably, concurrent use of FQs with other medications (i.e., combination therapy) exhibited the strongest signals [2.40 (2.31–2.49)], exceeding all FQ monotherapy (Figure 3A).

We further categorized PTs under High‐Level Group Terms (HLGTs), identifying “anxiety disorders and symptoms” as the most frequently reported psychiatric HLGT (3540 cases), followed by “sleep disorders and disturbances” (3236 cases), “depressed mood disorders and disturbances” (1967 cases), and “deliria (including confusion)” (1665 cases) (Figure 3B). Disproportionality analyses revealed four strong common safety signals reported by at least four FQs: anxiety disorders and symptoms [Overall ROR: 4.28 (4.13–4.43)], deliria (including confusion) [5.45 (5.19–5.72)], disturbances in thinking and perception [5.30 (5.00–5.60)], and sleep disorders and disturbances [4.97 (4.80–5.15)] (Figure 3C,D). Although positive signals for “suicidal and self‐injurious behaviours” [2.17 (2.02–2.33)] were detected in three FQs only, we included this in our analysis given its critical clinical significance in FQ‐associated psychiatric AEs. To identify previously unrecognized FQ‐associated psychiatric AEs, we first ranked PTs by occurrence frequency. The 20 most frequent PTs included both known AEs appearing in updated boxed warning labels and previously undescribed AEs (e.g., panic attack and emotional distress) (Figure S2A). Disproportionality analyses on the 50 most frequent PTs (Figure 3E) identified eight common PTs: anxiety [Overall ROR: 6.25 (6.00–6.51)], panic attack [17.11 (15.94–18.36)] (under “anxiety disorders and symptoms”), delirium [9.58 (8.71–10.54)] (under “deliria (including confusion)”), hallucination [6.65 (6.15–7.19)], hallucination, visual [6.58 (5.67–7.64)] (under “disturbances in thinking and perception”), insomnia [6.22 (5.96–6.49)], sleep disorder [6.24 (5.76–6.77)] (under “sleep disorders and disturbances”), and suicidal ideation [5.43 (5.02–5.87)] (under “suicidal and self‐injurious behaviors”) (Figure 3F).

**FIGURE 4:**
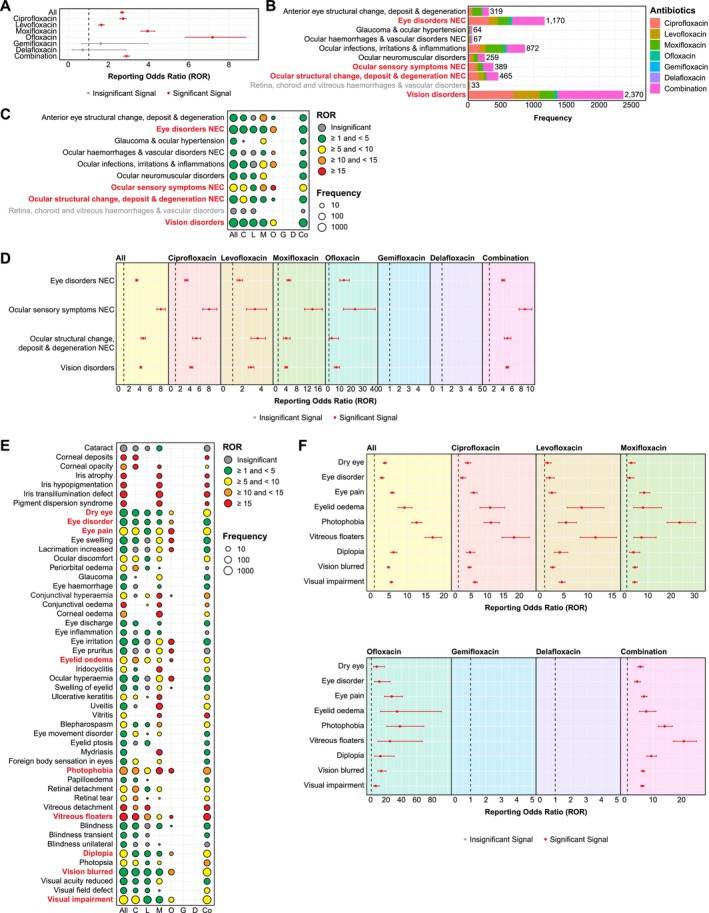
Identification of common and drug‐specific eye‐related AEs following FQ treatment using the FAERS database. (A) Disproportionality analysis revealed significant safety signals for eye‐related AEs associated with four FDA‐approved FQs: Ciprofloxacin, levofloxacin, moxifloxacin, and ofloxacin. (B) Each eye‐related PT was mapped with its primary HLGT. “Visual disorders” and “eye disorders” emerged as the most frequently reported eye‐related HLGTs following FQ treatment. (C) Heat map displaying ROR values of all eye‐related HLGTs. Dots represented HLGTs with at least three reported cases in FAERS, with dot sizes corresponding to their frequency for each FQ (C: ciprofloxacin; L: levofloxacin; M: moxifloxacin; O: ofloxacin; G: gemifloxacin; D: delafloxacin; Co: combination therapy). HLGT terms highlighted in red indicated common eye‐related positive safety signals reported by at least four FQs. (D) Forest illustrating ROR values (with 95% CI) of common eye‐related safety signals at HLGT levels reported by at least four FQs. Red denoted significant positive signals whilst gray indicated insignificant signals. (E) Heat map displaying ROR values of the top 50 common eye‐related PTs. Dots represented PTs with a minimum of three cases in FAERS, with dot sizes reflecting their frequency for each FQ. PTs highlighted in red denoted common safety signals observed reported with at least four FQs. (F) Forest plots illustrating ROR values (with 95% CI) of nine eye‐related PTs identified as common safety signals that were reported by at least four FQs. Red denoted significant positive signals whilst gray indicated insignificant signals.

Using the same methodology, we also calculated ROR values for each FQ, which revealed disproportionate reporting of eye‐related AEs in FQ‐associated AERs [Overall ROR: 2.66 (2.58–2.75)]. Of the six FDA‐approved FQs, positive safety signals were detected from four agents, with ofloxacin exhibiting the strongest signal [ROR: 7.16 (5.83–8.81)], followed by moxifloxacin [3.93 (3.61–4.28)], ciprofloxacin [2.70 (2.55–2.86)], and levofloxacin [1.62 (1.50–1.76)]. Combination therapies (involving two FQs, at least one FQ plus other antibiotics, or other medications) also demonstrated strong positive safety signals [2.87 (2.73–3.02)] (Figure 4A). Within all eye‐related HLGTs, “vision disorders” showed the highest frequency (2370 cases), followed by “eye disorders” (1170 cases) and “ocular infections, irritations and inflammations” (872 cases) (Figure 4B). Further disproportionality analyses revealed four common HLGTs with consistent safety signals detected across at least four FQs: eye disorders [Overall ROR: 3.46 (3.27–3.67)], ocular sensory symptoms [8.02 (7.25–8.87)], ocular structural changes, deposits and degeneration [4.67 (4.26–5.12)], and vision disorders [4.22 (4.05–4.40)] (Figure 4C,D).

Given the limited and inconsistent reports on FQ‐induced ocular toxicity (e.g., retinal detachment) [32, 33, 34, 35, 36], we sought to identify common FQ‐associated eye‐related safety signals. We first ranked each PT by occurrence frequency, identifying the 20 most frequently occurred PTs (Figure S2B). Subsequent disproportionality analyses on the 50 most frequent PTs (Figure 4E) identified nine common safety signals reported by at least four FQs: dry eye [Overall ROR: 3.89 (3.44–4.40)], eye disorder [3.06 (2.60–3.61)], eye pain [5.91 (5.38–6.49)] (under “eye disorders”), eyelid oedema [9.24 (7.53–11.35)] (under “ocular infections, irritations and inflammations”), photophobia [12.54 (11.21–14.03)] (under “ocular sensory symptoms”), vitreous floaters [16.96 (14.87–19.35)] (under “ocular structural change, deposit and degeneration”), diplopia [6.26 (5.47–7.16)], vision blurred [4.87 (4.55–5.21)], and visual impairment [5.67 (5.34–6.03)] (under “vision disorders”) (Figure 4F). With respect to retinal detachment, positive safety signals were observed for ciprofloxacin, levofloxacin, and moxifloxacin only (Figure 4E), with no AERs reporting retinal detachment associated with ofloxacin treatment in the FAERS database within the study period (2011–2024).

Additionally, we identified several moxifloxacin‐specific eye‐related AEs, including cataract [1.91 (1.17–3.12)] and corneal opacity [27.03 (11.22–65.12)]. Whilst isolated case reports have documented moxifloxacin‐associated iris abnormalities, we detected exceptionally high ROR values for iris atrophy [490.40 (283.73–847.60)], iris hypopigmentation [1887.66 (1175.44–3031.42)], iris transillumination defect [6604.89 (4736.76–9209.81)], and pigment dispersion syndrome [2360.62 (1432.71–3889.51)] (Figure 4E), signals that have not been previously reported using large‐scale pharmacovigilance data [37]. These moxifloxacin‐specific ocular abnormalities predominantly affected the anterior segment of the eye [ROR (anterior eye structural change, deposit and degeneration): 12.95 (11.00–15.25)] (Figure 4C). Pigment dispersion syndrome, the primary etiology of pigmentary glaucoma, may contribute to the positive safety signal for glaucoma [6.63 (4.17–10.53)] specific to moxifloxacin, suggesting a potential mechanistic pathway linking anterior segment toxicity to secondary glaucoma development [38, 39, 40]. Furthermore, we observed a strong moxifloxacin‐specific positive signal for mydriasis [15.81 (10.67–23.44)] (Figure 4E), which, together with pigmentary glaucoma, may result in irreversible visual impairment, highlighting the need for further clinical validation and mechanistic studies to better understand these ocular safety signals.

### 3.4 Time‐to‐Onset (TTO) Analysis Reveals a Fast Onset of Psychiatric and Eye‐Related AEs Following FQ Treatment

**FIGURE 5:**
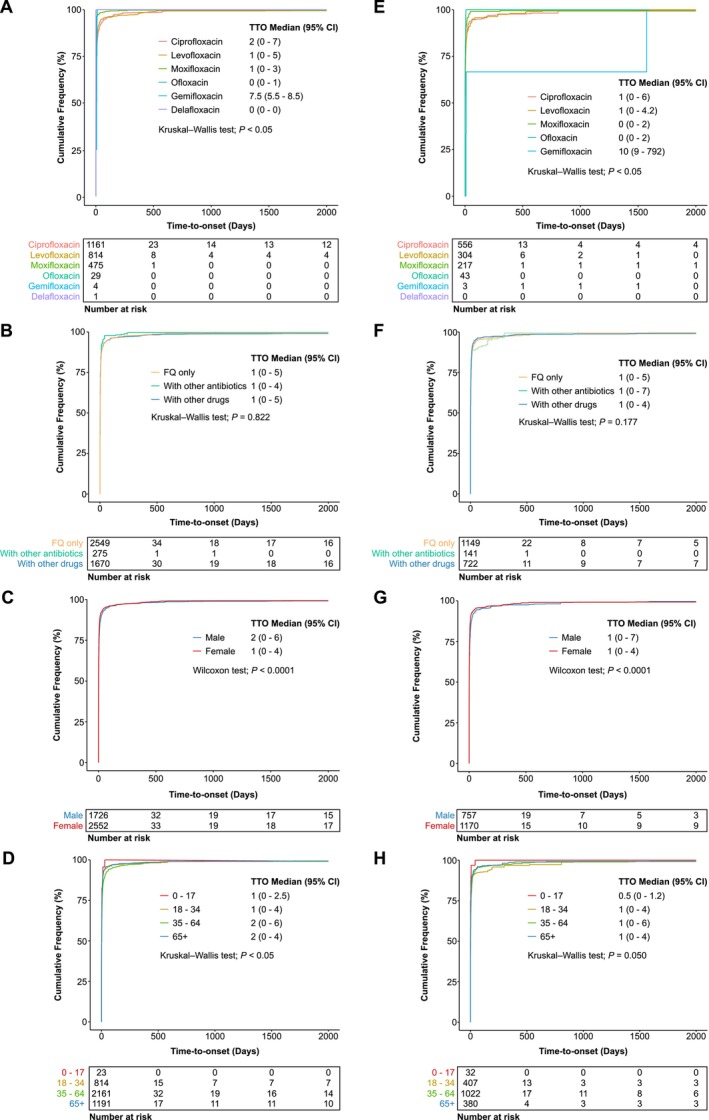
Time‐to‐onset (TTO) analyses on psychiatric and eye‐related AEs associated with FQ treatment. (A and E) Cumulative distribution curves showing the onset time of psychiatric (A) and eye‐related (E) AEs following treatment with individual FQs. (B and F) Cumulative distribution curves illustrating the onset time of psychiatric (B) and eye‐related (F) AEs in patients receiving FQ monotherapy versus combination therapy of at least one FQ with concurrent antibiotics or other medications. (C and G) Cumulative distribution curves demonstrating the onset time of psychiatric (C) and eye‐related (G) AEs across both sexes based on FAERS database. (D and H) Cumulative distribution curves illustrating the onset time of psychiatric (D) and eye‐related (H) AEs across different age groups. Statistical analyses were performed using the non‐parametric Kruskal–Wallis test in (A, B, D, E, F and H), and Wilcoxon rank sum test in (C and G).

In agreement with previous reports [15, 16], our time‐to‐onset (TTO) analysis of FQ‐associated psychiatric AERs confirmed that most psychiatric AEs typically occurred within 7 days of treatment initiation. The median onset time ranged from 0 to 2 days for most FQs, with the exception of gemifloxacin, which showed a delayed median onset of 7.5 days (Figure 5A). This pattern remained largely unchanged whether FQs were administered as monotherapy or in combination with other antibiotics or medications (Figure 5B). Notably, females exhibited a modest yet significantly earlier onset time [median: 1 (0–4)] compared to males [2 (0–6)] (Figure 5C). A similar trend was observed across age groups, with younger individuals (aged 0–17 and 18–34 years) experiencing slightly earlier onset than older populations (aged 35–64 and ≥ 65 years) (Figure 5D).

A similar pattern was observed in the TTO analysis of FQ‐associated eye‐related AEs. As with psychiatric AEs, most eye‐related AEs appeared within 7 days of FQ initiation, except for gemifloxacin (Figure 5E). No significant difference in the TTO pattern of eye‐related AEs was observed when FQs were administered as monotherapy or in combination with other antibiotics or drugs (Figure 5F). Interestingly, while both sexes showed a median onset of 1 day, a higher percentage of females (82.82%) developed eye‐related AEs within 7 days, compared with males (74.77%) (Figure 5G). Median onset times across all age groups were similar, ranging narrowly from 0.5 to 1 day following FQ treatment (Figure 5H).

### 3.5 Factors Influencing FQ‐Associated Psychiatric AEs

**FIGURE 6:**
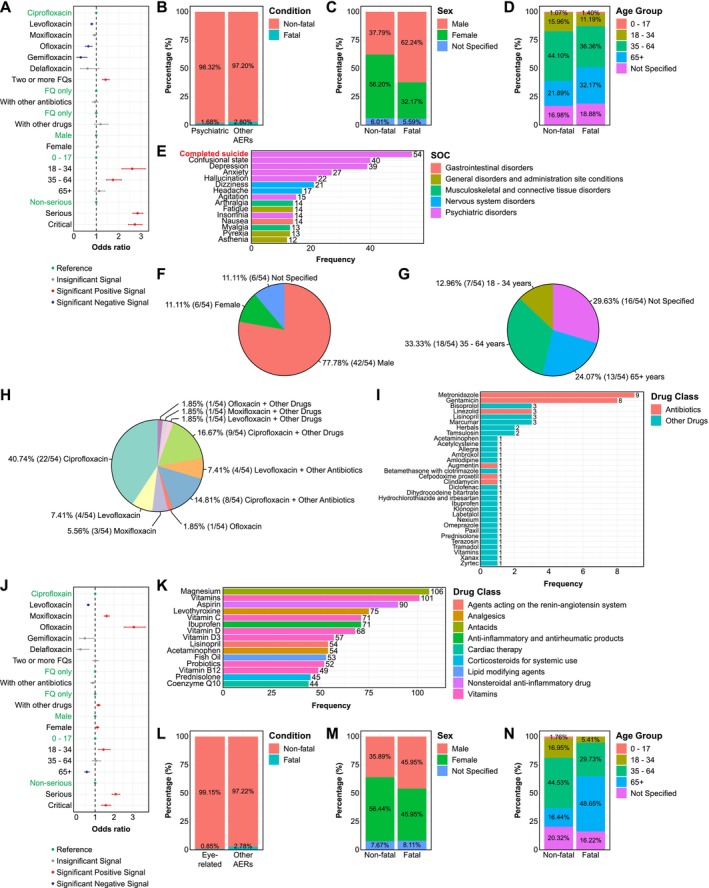
Factor influencing psychiatric and eye‐related AEs associated with FQ treatment. (A) Forest plot summarizing univariate logistic regression analyses of factors influencing the reporting of psychiatric AEs associated with FQ treatment. Red denoted significant positive signals, whilst blue indicated significant negative signals. Gray represented insignificant signals, and green indicated reference factors. (B) FQ‐associated AERs with psychiatric AEs demonstrated substantially lower proportion of fatal outcomes compared with those exhibiting other AEs (Chi‐square test; *p* = 5.02 × 10^−9^). (C) Males accounted for the majority of FQ‐associated fatal psychiatric AERs, whilst females predominated in non‐fatal psychiatric AERs (Chi‐square test; *p* = 1.13 × 10^−8^). (D) A significantly higher proportion of elderly individuals (≥ 65 years) was observed among fatal psychiatric AERs compared with non‐fatal cases (Fisher's exact test; *p* = 0.0254). (E) “Completed suicide” emerged as the most frequently reported PT among all AEs in FQ‐associated fatal psychiatric AERs, accounting for 58 cases. (F and G) Among all FQ‐associated fatal psychiatric AERs involving suicide, the majority were males (F) aged 35–64 years (G). (H) Ciprofloxacin was the most frequently implicated FQ among fatal psychiatric AERs involving suicide. (I) For combination therapy, metronidazole and gentamicin were among the most frequently reported concomitant medications with FQs in fatal psychiatric AERs involving suicide. (J) Forest plot displaying univariate logistic regression analysis of factors influencing the reporting of eye‐related AEs. Red denoted significant positive signals, whilst blue indicated significant negative signals. Gray represented insignificant signals, and green indicated reference factors. (K) Bar plot depicting the most commonly reported concomitant medications with FQs in eye‐related AERs. (L) FQ‐associated AERs with eye‐related AEs exhibited substantially lower proportion of fatal outcomes compared with those displaying other AEs (Chi‐square test; *p* = 3.39 × 10^−14^). (M) The distribution of fatal eye‐related AERs was similar between males and females, but there was no statistically significant sex difference between fatal and non‐fatal cases (Fisher's exact test; *p* = 0.401). (N) FQ‐associated fatal eye‐related AERs showed a substantially higher proportion of elderly patients (≥ 65 years) compared with non‐fatal cases (Fisher's exact test; *p* = 2.21 × 10^−4^).

To further explore potential factors influencing the reporting of psychiatric AEs associated with FQ use, we performed univariate logistic regression and calculated odds ratios (ORs) based on all FQ‐associated AERs [20]. With ciprofloxacin as the reference, three FQ demonstrated substantially lower ORs for psychiatric AEs: levofloxacin [OR: 0.80 (0.76–0.85)], ofloxacin [0.65 (0.51–0.81)], and gemifloxacin [0.31 (0.14–0.58)], whilst moxifloxacin [0.93 (0.86–1.00)] and delafloxacin [0.61 (0.30–1.13)] did not differ significantly from ciprofloxacin (*p* > 0.05) (Figure 6A). Notably, combination therapy with multiple FQs showed 1.42‐fold higher odds of psychiatric AEs [OR: 1.42 (1.27–1.58)] compared with FQ monotherapy, whereas combining FQs with other antibiotics or medications had minimal impact on the oddsof psychiatric AEs (Figure 6A). Sex did not emerge as an influential factor, with both males and females showing similar odds of developing psychiatric AEs following FQ treatment. However, both young adults (18–34 years) and middle‐aged individuals (35–64 years) exhibited substantially higher odds compared with children and adolescents (0–17 years), with ORs of 2.60 (2.14–3.19) and 1.75 (1.45–2.13), respectively, whilst no significant difference was observed in the elderly (≥ 65 years) [1.14 (0.94–1.39)] (Figure 6A). Importantly, patients experiencing serious [OR: 2.84 (2.63–3.08)] or critical (life‐threatening or fatal) [2.72 (2.43–3.05)] adverse reactions demonstrated significantly higher odds of developing psychiatric AEs following FQ treatment compared with those experiencing non‐serious outcomes (Figure 6A).

### 3.6 Mortality Patterns in FQ‐Associated AEs

Throughout the entire study period (2011–2024), 1163 fatal cases were documented following FQ treatment, comprising 2.59% of total FQ‐associated AERs (Figure S3A). Whilst females predominated in the overall population of FQ‐associated AERs, males constituted the majority of fatal cases [47.12% (548/1163)], with most fatalities reported in elderly populations (≥ 65 years) [41.62% (484/1163)] (Figure S3B,C). Among these fatal cases, the most frequently reported AEs were drug ineffectiveness (92/1163), cardiac arrest (59/1163), and completed suicide (54/1163) (Figure S3D).

Our disproportionality analysis identified suicidal ideation as a common safety signal for FQ‐associated psychiatric AEs, with completed suicide ranking among one of the most frequently reported AEs in fatal cases. This prompted further investigations into potential associations between FQ‐associated psychiatric AEs and mortality. Interestingly, only 1.68% (143/8518) of FQ‐associated psychiatric AERs resulted in fatal outcomes, compared with 2.80% (1020/36 377) of FQ‐associated AERs without psychiatric manifestations (Figure 6B). Of the 143 fatal cases with psychiatric AEs, 62.24% (89/143) were males (Figure 6C), with the majority [36.36% (52/143)] being middle‐aged individuals (35–64 years) (Figure 6D).

Notably, “completed suicide” was the most frequently reported AE in these fatal psychiatric cases, accounting for 37.76% (54/143) (Figure 6E), indicating that suicide was likely the predominant cause of fatality in FQ‐associated psychiatric AERs. Strikingly, we observed a potential gender paradox in FQ‐associated suicidal behaviors that was previously uncharacterized [17]: whilst males comprised 62.24% of fatal psychiatric cases overall (Figure 6C), they constituted a disproportionate 77.78% (42/54) of individuals who died by suicide following FQ treatment, compared with only 11.11% (6/54) females (Figure 6F). This male predominance in completed suicide contrasted markedly with the broader pattern observed in FQ‐associated psychiatric AERs, where females were typically over‐represented (Table 1). The most frequently represented age group was middle‐aged individuals (35–64 years), accounting for 33.33% (18/54) of suicidal cases, followed by young adults (18–34 years) [29.63% (16/54)] and the elderly (≥ 65 years) [24.07% (13/54)] (Figure 6G). Ciprofloxacin was the most frequently implicated FQ in these suicide cases (Figure 6H), either as monotherapy [40.74% (22/54)] or in combination with other medications [16.67% (9/54)] or antibiotics [14.81% (8/54)], such as metronidazole and gentamicin (Figure 6I). Suicidal cases were also detected following treatment with levofloxacin, moxifloxacin, and ofloxacin, but not with gemifloxacin or delafloxacin (Figure 6H).

### 3.7 Factors Influencing FQ‐Associated Eye‐Related AEs and Their Mortality Patterns

We also conducted univariate logistic regression and calculated ORs using all FQ‐associated AERs to identify factors influencing the reporting of eye‐related AEs following FQ treatment. Taking ciprofloxacin as the reference, moxifloxacin [OR: 1.61 (1.47–1.75)] and ofloxacin [3.06 (2.54–3.66)] demonstrated substantially higher odds of developing eye‐related AEs, whereas levofloxacin [0.63 (0.58–0.68)] exhibited relatively lower odds. Gemifloxacin [0.45 (0.18–0.95)] and delafloxacin [0.21 (0.03–0.66)] did not show significant differences in ORs compared with ciprofloxacin (*p* > 0.05) (Figure 6J). Whilst concurrent use of two or more FQs did not increase the OR [1.01 (0.87–1.17)] compared with FQ monotherapy, combining FQs with other medications increased the odds of developing eye‐related AEs by 1.17 times [1.17 (1.09–1.26)] (Figure 6J), with the most frequently co‐administered medications with FQs presenting in Figure 6K. Compared with males, females demonstrated a modest yet significant increase in odds [1.12 (1.04–1.19)] of developing eye‐related AEs following FQ treatment (Figure 6J). Age also influenced reporting patterns: young adults (18–34 years) exhibited a significantly higher odds of eye‐related AEs following FQ treatment [1.44 (1.17–1.80)] when compared with children and adolescents (0–17 years), whereas the elderly (≥ 65 years), unexpectedly, showed relatively lower odds [0.55 (0.45–0.69)] (Figure 6J). Similarly, the severity of the outcome was another important influencing factor: patients experiencing serious [2.09 (1.90–2.31)] or critical [1.56 (1.34–1.82)] outcomes displayed considerably higher odds of eye‐related AEs than those with non‐serious outcomes (Figure 6J).

Among all FQ‐associated AERs displaying eye‐related AEs, 37 cases were fatal, representing 0.85% of all FQ‐associated eye‐related AERs (Figures 6L and S4A). The gender distribution for these fatal cases was balanced, with 17 cases each for males and females, each accounting for 45.95% of these fatal events (Figure 6M). Most fatal cases occurred in the elderly (≥ 65 years), accounting for 48.65% (18/37) of all fatal FQ‐associated eye‐related AERs (Figure 6N). The most frequently reported AE among these fatal cases was “visual impairment”, an eye‐related adverse reaction. Interestingly, the majority of concurrent AEs in those fatal FQ‐associated AERs with eye‐related AEs involved non‐ocular systems, suggesting potential systemic complications or multi‐organ involvement in these severe and fatal cases (Figure S4B).

## 4 Discussion

Whilst being one of the most widely prescribed antibiotics globally for treating numerous bacterial infections [11, 41], FQs have raised substantial safety concerns regarding their disabling and potentially permanent side effects on tendons, muscles, peripheral nerves and CNS, prompting various revisions of boxed warnings by the FDA in the recent decade. Our current study provided a comprehensive global pharmacovigilance analysis of FQ‐associated adverse reactions using real‐world data from the FAERS database, identifying eight psychiatric and nine eye‐related AEs commonly reported following FQ treatment. Several of these AEs were not previously documented in the literature. Notably, suicide was the most frequently reported fatal outcome among reports of FQ‐associated psychiatric AERs. Despite major regulatory agencies including the FDA, EMA, MHRA and TGA advising restricted use of FQs in uncomplicated bacterial infections [13], our descriptive analysis revealed no declining trend in AERs, with numbers remaining relatively constant throughout the study period. This suggests the potential need for more stringent restrictions, implementation of robust safety monitoring programmes, as well as guidelines focused on early detection and intervention for FQ‐associated psychiatric and eye‐related AEs.

Accumulating evidence suggests that FQ treatment induces adverse reactions in both PNS and CNS [14]. FQs are known to cause peripheral neuropathy [42], likely due to the relative vulnerability of peripheral nerves to neurotoxic agents (e.g., FQs or chemotherapeutic agents) caused by the less protective blood‐nerve barrier [43]. Notably, FQ‐induced peripheral neuropathy can be permanent and irreversible, potentially due to ineffective nerve repair mechanisms following FQ exposure [44, 45]. In the CNS, FQs induce psychiatric AEs owing to their effective penetration into the cerebrospinal fluid [46]. FQ‐associated psychiatric AEs tend to resolve completely within days or weeks following drug discontinuation [47, 48, 49, 50]. Mechanistically, FQs are known to bind to GABA_A_ receptors with high affinity, antagonizing the action of their natural ligand (i.e., GABA) as the primary inhibitory neurotransmitter in the CNS [51]. This GABAergic disruption is believed to underlie CNS hyperexcitability, leading to the emergence of anxiety or panic attacks following FQ treatment [52]. Additionally, the disturbances in other neurotransmitter systems, including dopamine and glutamate [53], may further contribute to acute psychiatric manifestations such as delirium [54] and hallucinations [55]. Whilst most common psychiatric positive signals for FQs identified in the current study were already included in the revised box label warning, panic attack—a novel adverse reaction not documented in the revised labels—might serve as an excellent early clinical indicator for the onset of more severe mental health conditions associated with FQ treatment (e.g., hallucinations or suicidal attempts) and could robustly help with safety monitoring of patients following FQ therapies [56, 57]. Importantly, conventional antipsychotics appear ineffective for managing FQ‐induced psychiatric AEs such as psychosis and delirium. Instead, complete withdrawal of the implicated FQ generally leads to full resolution of psychiatric symptoms [15], underscoring the importance of early recognition and prompt discontinuation. In such cases, clinicians should prioritize discontinuing the offending FQs and promptly explore alternative antimicrobial options with the patients, instead of prescribing antipsychotic medications after the first onset of FQ‐associated psychiatric AEs.

Whilst FDA warnings acknowledge psychiatric side effects including mental health disturbances associated with FQ use, systematic characterization of demographic patterns and identification of high‐risk subgroups for FQ‐associated suicide have been lacking. Our current study revealed critical patterns of FQ‐associated suicidal behavior that might inform clinical awareness and hypothesis generation for future risk stratification studies. Although suicide represented a numerically rare event [0.63% (54/8518)] among all FQ‐associated psychiatric AEs, consistent with previous reports suggesting occurrence in less than 1% of treated patients [58], it accounted for the majority [37.76% (54/143)] of fatal outcomes in individuals with FQ‐associated psychiatric AEs. This disproportionate representation among fatalities underscored the catastrophic nature of this AE and its significance in clinical practice. Importantly, our analysis identified distinct demographic vulnerability patterns. We observed a striking gender paradox: whilst females compromised the majority of psychiatric AE cases overall (55.80%), males accounted for 77.78% of completed suicides following FQ treatment, compared with only 11.11% for females. Additionally, our analysis revealed that middle‐aged individuals (35–64 years) represented the highest proportion of these AERs [33.33% (18/54)], followed by young adults (18–34 years) [29.63% (16/54)] and the elderly (≥ 65 years) [24.07% (13/54)]. These findings raised the possibility that the development of mild psychiatric symptoms might escalate within days to suicide attempts if not recognized and managed promptly [59]. Furthermore, whilst suicidal ideation is listed as a potential adverse reaction for most FDA‐approved FQs (ciprofloxacin, levofloxacin, ofloxacin, and delafloxacin), it is notably absent from the product label of Avelox (moxifloxacin) [58]. Given that suicidal ideation emerged as one of the common safety signals reported by major FQs in our disproportionality analysis (ROR: 5.43), this inconsistency in product labelling highlights the need for re‐evaluation, particularly for moxifloxacin, where psychiatric risks may be under‐recognized. Collectively, our findings provided novel, real‐world pharmacovigilance evidence on the demographic determinants of FQ‐associated suicidal behaviors and underscored the importance of enhanced clinical vigilance, particularly for middle‐aged male patients, following FQ treatment.

The ability of FQs to effectively penetrate the blood‐retinal barrier and achieve high intraocular concentrations makes them valuable agents for treating ocular infections [60]. Adverse ocular effects, including dry eye, photophobia, and blurred vision, have been reported more frequently following ophthalmic rather than systemic administration of FQs, as noted in product labelling. Whilst FQs have historically been regarded as having low ocular toxicity [34, 61], preclinical investigations suggest they may induce retinal degeneration or retinopathy [62, 63, 64]. Given their known photosensitising properties [65], it is plausible that FQs could elicit photophobia and other light‐related ocular symptoms in humans [66]. This photosensitivity may also contribute to secondary adverse effects such as eyelid oedema—one of the common safety signals identified in our analysis—which could result from drug hypersensitivity, with photosensitivity acting as a compounding factor [67]. In clinical settings, retinal detachment has been flagged as a serious ocular adverse effect associated with FQ treatment that might result in permanent visual impairment. However, findings across studies remain inconclusive [32, 33, 35, 36], likely due to methodological differences and inherent limitations of the study designs [68]. In our analysis, retinal detachment did not emerge as a common safety signal across the most widely used FQs, with ofloxacin standing out as the only agent for which no associated AERs were identified throughout the entire study period. Interestingly, vitreous floaters emerged as a positive safety signal reported across FDA‐approved FQs, with noticeably high ROR values. The occurrence of vitreous floaters, alongside blurred vision (another common positive safety signal in our analysis), may serve as early clinical indicators for healthcare professionals regarding potential subsequent retinal detachment following FQ treatment [69]. Among the six FDA‐approved FQs included in our analysis, ofloxacin demonstrated the highest ROR values for eye‐related AEs, with a remarkable 22.58% of all ofloxacin‐associated AERs involving ocular manifestations. This observation might be partially attributed to its widespread use in ophthalmic preparations [70], facilitated by its superior ocular bioavailability (10‐fold greater than ciprofloxacin) [60], where direct contact with ocular tissues at high concentrations may heighten the risk of localized adverse side effects. Since our data retrieved from the FAERS Public Dashboard lacked information on administration routes, it was not possible to determine whether the ofloxacin‐associated eye‐related AEs arose from systemic or ophthalmic administration. Given this limitation, further investigation into the ocular safety profiles of individual FQs is therefore warranted to elucidate mechanisms underlying FQ‐induced ocular toxicity and thereby better inform clinical risk assessment and patient monitoring strategies.

In the current study, we detected exceptionally high safety signals for ocular AEs associated with moxifloxacin (ROR: 3.93) and ofloxacin (ROR: 7.16) (Figure 4A). While ofloxacin demonstrated the strongest overall signal, the ocular AEs detected were shared among multiple FQs rather than being ofloxacin‐specific (Figure 4E). In contrast, moxifloxacin exhibited substantial positive safety signals for various ocular AEs predominantly affecting the anterior segment, including the cornea and iris (Figure 4C). While isolated case reports have previously documented iris depigmentation and transillumination following moxifloxacin treatment [71, 72, 73], our current study provided the first large‐scale quantification of these events using real‐world data, revealing strikingly high reporting odds ratios for iris transillumination defect (ROR: 6604.89), iris hypopigmentation (ROR: 1887.66), and iris atrophy (ROR: 490.40) (Figure 4E). Notably, moxifloxacin also demonstrated a substantial positive safety signal for pigment dispersion syndrome (ROR: 2360.62) (Figure 4E), a condition that may lead to pigment accumulation in the trabecular meshwork and is recognized as a key etiology of pigmentary glaucoma [74]. Consistent with this observation, glaucoma itself emerged as a moxifloxacin‐specific positive safety signal (Figure 4E), aligning with a recent study reporting pigmentary degeneration of the iris and pigmentary glaucoma in patients receiving moxifloxacin but not levofloxacin [75]. Beyond glaucoma, our analysis identified additional moxifloxacin‐specific signals for vision‐impairing conditions including mydriasis (abnormal pupil dilation), corneal opacity, and cataract (Figure 4E). Although the mechanisms underlying these associations remain unclear, in vitro evidence suggested moxifloxacin exhibits dose‐dependent neurotoxicity to retinal ganglion cells [76], which may contribute to the visual impairment signals observed in our analysis [77, 78, 79]. Given that both mydriasis [80] and pigmentary glaucoma [75] associated with moxifloxacin use may result in irreversible visual impairment, our findings highlight the need for further investigation into the ocular safety profile of moxifloxacin. Importantly, these moxifloxacin‐specific ocular safety signals are not prominently featured in current FDA black box warnings, suggesting a potential gap in risk communication that merits consideration for enhanced product labelling and clinical monitoring strategies.

This FAERS‐based pharmacovigilance study has several inherent limitations. Underreporting represents the most significant constraint, particularly among elderly populations (≥ 65 years) who may encounter barriers to accessing direct online reporting systems, potentially leading to underestimation of FQ‐associated psychiatric and ocular risks in this vulnerable population. Furthermore, critical confounding variables such as dosage regimens and medical history were either missing or incompletely documented in the FAERS Public Dashboard. Our initial objective was to identify potential psychiatric and eye‐related AEs following treatment with each FDA‐approved FQ. However, we were unable to detect any specific AEs for gemifloxacin and delafloxacin, largely attributable to insufficient sample sizes within the study timeframe. This limitation likely stems from their limited clinical utilization, potentially due to their heightened cost compared with more widely prescribed FQs such as ciprofloxacin and levofloxacin [81]. Additionally, since delafloxacin only received FDA approval in 2017, the current dataset may not yet reflect the full range of psychiatric and eye‐related AEs associated with its use. Nonetheless, our study relied solely on a single database (FAERS). To enhance signal robustness, future studies should aim to confirm our findings using independent pharmacovigilance databases such as VigiBase [23]. To overcome these limitations, large‐scale multinational retrospective cohort or case–control studies leveraging electronic health records are warranted to comprehensively characterize the entire spectrum of psychiatric and eye‐related adverse reactions associated with each FQ, including newer agents such as gemifloxacin and delafloxacin.

In summary, our current study using real‐world data from the FAERS database identified substantial safety signals for psychiatric and eye‐related AEs associated with FQ treatment. This finding is particularly concerning given that FQs remain among the most widely prescribed antibiotics globally. Whilst major regulatory agencies such as the FDA, EMA, MHRA, and TGA have issued explicit guidelines urging healthcare professionals to limit FQ prescriptions to cases where no suitable alternatives exist, some countries (e.g., Hong Kong SAR) have merely reiterated these announcements from these regulatory agencies without providing specific instructions or clear clinical protocols for local practitioners regarding FQ use [82]. More problematically, in certain countries (e.g., Thailand, India, and many developing countries), FQs remain readily available with clinicians able to prescribe them without restrictions [11, 83, 84]. This global inconsistency in regulatory policy underscores an urgent need for a unified, evidence‐based antimicrobial stewardship strategy that not only reduces the overuse of FQ antibiotics [85] but also reinforces their use strictly as a last‐line treatment option when other antibiotics are inappropriate [86]. When FQ prescription is deemed unavoidable, effective patient post‐prescription monitoring systems (e.g., mobile electroencephalogram devices for tracking drug‐induced psychiatric events [87, 88, 89], or smartphone applications enabling patients to promptly report adverse events to clinicians [90]) should be implemented. These tools can facilitate the early identification of rapidly emerging neuropsychiatric and ocular complications, enabling timely discontinuation of these agents and minimizing the risk of potentially irreversible harm. Ultimately, a combined approach incorporating regulatory vigilance, informed prescribing practices, and proactive pharmacovigilance will be critical to safeguarding patient safety whilst preserving the efficacy of this important, yet potentially hazardous, class of antibiotics.

## Author Contributions

**Hau‐Tak Chau:** investigation, formal analysis, visualization. **Ngan Pan Bennett Au:** funding acquisition, writing – original draft, methodology, writing – review and editing, data curation, formal analysis, supervision, investigation, project administration.

## Conflicts of Interest

The authors declare no conflicts of interest.

## Supporting information


**Figure S1:** Flow chart depicting the selection process of adverse event reports (AERs) associated with fluoroquinolone (FQ) treatment. The complete dataset of AERs involving six FDA‐approved FQs (ciprofloxacin, levofloxacin, moxifloxacin, ofloxacin, gemifloxacin and delafloxacin) as potential suspects was extracted from the FDA Adverse Event Reporting System (FAERS) Public Dashboard, 2011–2024. Following deduplication and exclusion of concomitant use of drugs known to induce psychiatric adverse events (those classified under Anatomical Therapeutic Chemical codes N05 Psycholeptics, N06 Psychoanaleptics, and N07B Drugs Used in Addictive Disorders), a total of 44 895 cases were retained for subsequent descriptive and disproportionality analyses.
**Figure S2:** The 20 most frequently preferred terms (PTs) in FQ‐associated AERs with psychiatric and eye‐related adverse events (AEs). (A) Each PT with “psychiatric disorders” as the primary System Organ Class (SOC) was initially mapped to its High Level Group Term (HLGT) and the frequency of each psychiatric PT was determined from all FQ‐associated AERs with psychiatric AEs. The bar plot illustrates the 20 most common psychiatric AEs, with colors indicating their primary HLGT. (B) Similarly, each PT with “eye disorders” as the primary SOC was similarly mapped to its HLGT and the frequency of each eye‐related PT was determined from all FQ‐associated AERs with eye‐related AEs. The bar plot presents the 20 most common eye‐related AEs, with colors denoting their primary HLGT.
**Figure S3:** Characteristics of fatal cases associated with FQ treatment. (A) Throughout the entire study period, a total of 1163 fatal cases were identified, representing 2.59% of total AERs associated with FQ treatment. (B) Whilst females constituted the majority of FQ associated non‐fatal AERs, males predominated in the FQ‐associated fatal AERs (Chi‐square test; *p* = 8.41 × 10–29). (C) Fatal cases were more prevalent in the elderly population (≥ 65 years) compared with non‐fatal AERs (Chi‐square test; *p* = 1.64 × 10–34). (D) Among these FQ associated fatal AERs, the three most commonly reported AEs were drug ineffective, cardiac arrest and completed suicide. Bar colors indicate the primary SOC of each PT.
**Figure S4:** Characteristics of FQ‐associated fatal cases with eye‐related AEs. (A) Throughout the entire study period, a total of 37 fatal cases were identified, comprising 0.85% of total FQ associated AERs with eye‐related AEs. (B) Among these fatal cases, the three most frequently reported AEs were visual impairment, confusional state and headache. Bar colors indicate the corresponding primary SOC.
**Table S1:** Annual case counts of adverse event reports (AERs) associated with fluoroquinolone (FQ) treatment, based on data from the FDA Adverse Event Reporting System (FAERS) Public Dashboard, 2011–2024.
**Table S2:** Demographic characteristics of adverse event reports (AERs) associated with fluoroquinolone (FQ) treatment, based on FAERS data, 2011–2024.

## Data Availability

All data generated or analyzed in this study are included in the manuscript and its Supporting Information. All AERs are publicly available and can be retrieved directly from the FAERS Public Dashboard (https://www.fda.gov/drugs/fdas‐adverse‐event‐reporting‐system‐faers/fda‐adverse‐event‐reporting‐system‐faers‐public‐dashboard).
