# Supplementary material for: Fluoroquinolone‐Associated Psychiatric and Ocular Adverse Events: A Disproportionality Analysis Using Real‐World Data From FAERS (2011–2024)
Source: Pharmacol Res Perspect. 2025 Dec 17;14(1):e70206. doi: 10.1002/prp2.70206 (PMC12712350; doi:10.1002/prp2.70206)
Supplement: Supplementary file 1 — Figure S1: Flow chart depicting the selection process of adverse event reports (AERs) associated with fluoroquinolone (FQ) treatment. The complete dataset of AERs involving six FDA‐approved FQs (ciprofloxacin, levofloxacin, moxifloxacin, ofloxacin, gemifloxacin and delafloxacin) as potential suspects was extracted from the FDA Adverse Event Reporting System (FAERS) Public Dashboard, 2011–2024. Following deduplication and exclusion of concomitant use of drugs known to induce psychiatric adverse events (those classified under Anatomical Therapeutic Chemical codes N05 Psycholeptics, N06 Psychoanaleptics, and N07B Drugs Used in Addictive Disorders), a total of 44 895 cases were retained for subsequent descriptive and disproportionality analyses. Figure S2: The 20 most frequently preferred terms (PTs) in FQ‐associated AERs with psychiatric and eye‐related adverse events (AEs). (A) Each PT with “psychiatric disorders” as the primary System Organ Class (SOC) was initially mapped to its High Level Group Term (HLGT) and the frequency of each psychiatric PT was determined from all FQ‐associated AERs with psychiatric AEs. The bar plot illustrates the 20 most common psychiatric AEs, with colors indicating their primary HLGT. (B) Similarly, each PT with “eye disorders” as the primary SOC was similarly mapped to its HLGT and the frequency of each eye‐related PT was determined from all FQ‐associated AERs with eye‐related AEs. The bar plot presents the 20 most common eye‐related AEs, with colors denoting their primary HLGT. Figure S3: Characteristics of fatal cases associated with FQ treatment. (A) Throughout the entire study period, a total of 1163 fatal cases were identified, representing 2.59% of total AERs associated with FQ treatment. (B) Whilst females constituted the majority of FQ associated non‐fatal AERs, males predominated in the FQ‐associated fatal AERs (Chi‐square test; p = 8.41 × 10^–29^). (C) Fatal cases were more prevalent in the elderly population (≥ 65 years) compar [file PRP2-14-e70206-s001.pdf]

Supplementary Information

Fluoroquinolone-associated psychiatric and ocular adverse events: a disproportionality analysis using real-world data from FAERS (2011-2024)

Hau-Tak Chau[1], Ngan Pan Bennett Au[2,*]

[1] Department of Medicine, School of Clinical Medicine, The University of Hong Kong, Hong Kong SAR.

[2] Department of Comparative Biomedical Sciences, School of Veterinary Medicine, University of Surrey, Guildford GU2 7AL, UK.

Running title: Fluoroquinolone-induced psychiatric and eye disorders

* Correspondence: Dr. Ngan Pan Bennett Au

[2] Department of Comparative Biomedical Sciences, School of Veterinary Medicine, University of Surrey, Guildford GU2 7AL, UK.

Email: n.au@surrey.ac.uk

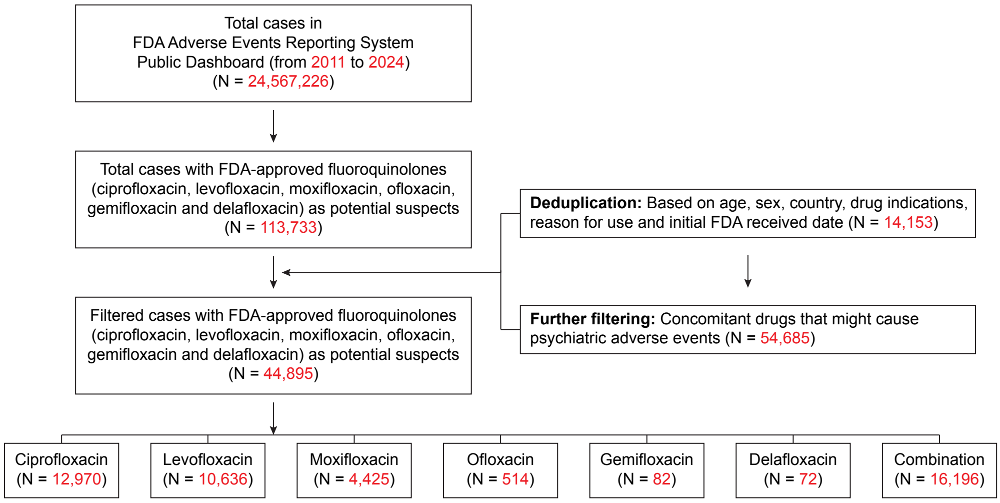

Fig. S1. Flow chart depicting the selection process of adverse event reports (AERs) associated with fluoroquinolone (FQ) treatment. The complete dataset of AERs involving six FDA-approved FQs (ciprofloxacin, levofloxacin, moxifloxacin, ofloxacin, gemifloxacin and delafloxacin) as potential suspects was extracted from the FDA Adverse Event Reporting System (FAERS) Public Dashboard, 2011 - 2024. Following deduplication and exclusion of concomitant use of drugs known to induce psychiatric adverse events (those classified under Anatomical Therapeutic Chemical codes N05 Psycholeptics, N06 Psychoanaleptics, and N07B Drugs Used in Addictive Disorders), a total of 44,895 cases were retained for subsequent descriptive and disproportionality analyses.

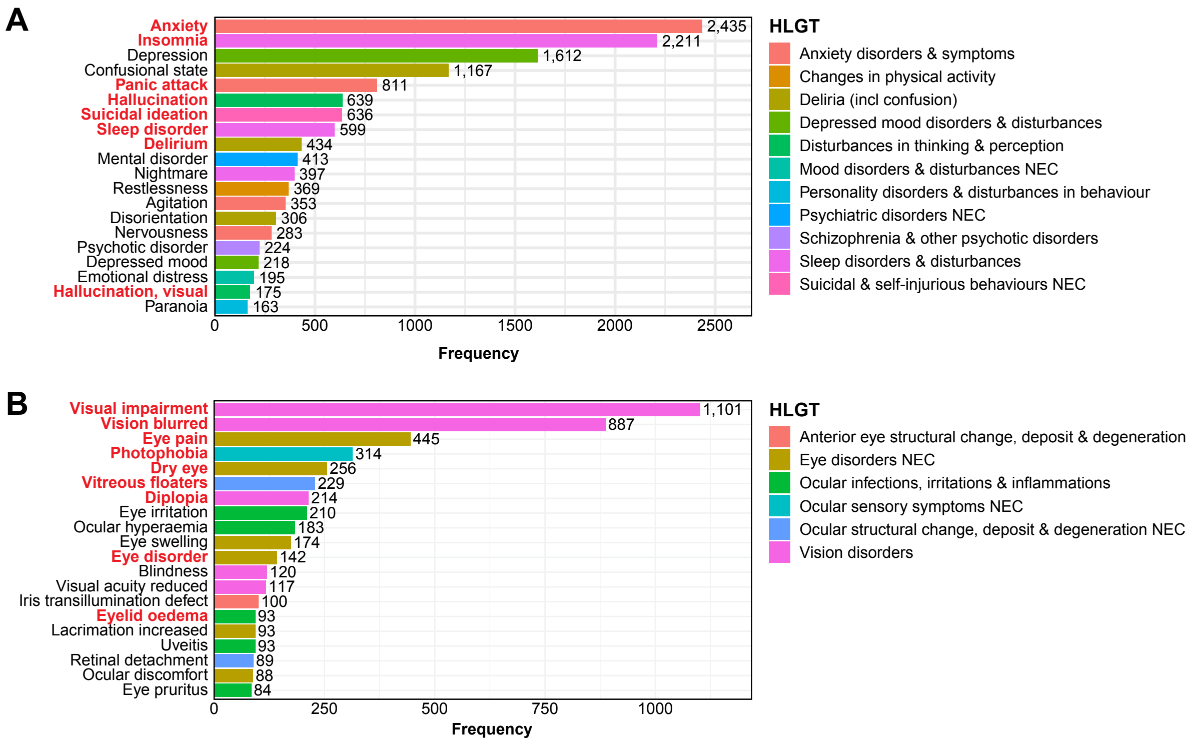

Fig. S2. The 20 most frequently preferred terms (PTs) in FQ-associated AERs with psychiatric and eye-related adverse events (AEs). (A) Each PT with "psychiatric disorders" as the primary System Organ Class (SOC) was initially mapped to its High Level Group Term (HLGT) and the frequency of each psychiatric PT was determined from all FQ-associated AERs with psychiatric AEs. The bar plot illustrates the 20 most common psychiatric AEs, with colours indicating their primary HLGT. **(B)** Similarly, each PT with "eye disorders" as the primary SOC was similarly mapped to its HLGT and the frequency of each eye-related PT was determined from all FQ-associated AERs with eye-related AEs. The bar plot presents the 20 most common eye-related AEs, with colours denoting their primary HLGT.

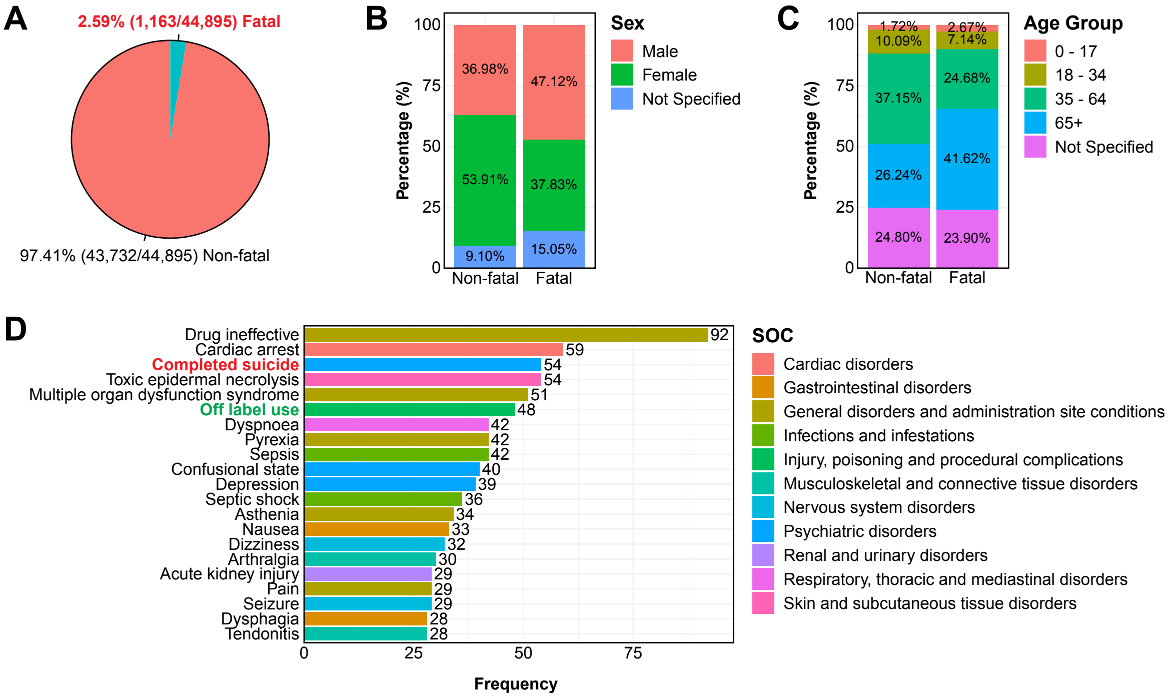

Fig. S3. Characteristics of fatal cases associated with FQ treatment. (A) Throughout the entire study period, a total of 1,163 fatal cases were identified, representing 2.59% of total AERs associated with FQ treatment. **(B)** Whilst females constituted the majority of FQ-associated non-fatal AERs, males predominated in the FQ-associated fatal AERs (Chi-square test; $P = 8.41 \times 10^{-29}$). **(C)** Fatal cases were more prevalent in the elderly population (≥65 years) compared with non-fatal AERs (Chi-square test; $P = 1.64 \times 10^{-34}$). **(D)** Among these FQ-associated fatal AERs, the three most commonly reported AEs were drug ineffective, cardiac arrest and completed suicide. Bar colours indicate the primary SOC of each PT.

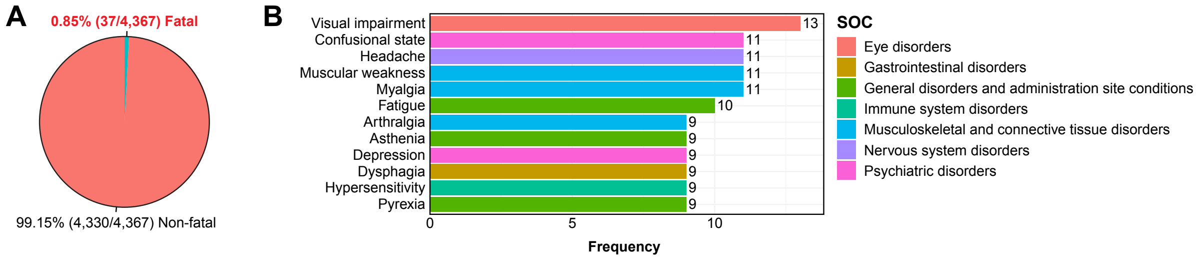

Fig. S4. Characteristics of FQ-associated fatal cases with eye-related AEs. (A) Throughout the entire study period, a total of 37 fatal cases were identified, comprising 0.85% of total FQ-associated AERs with eye-related AEs. **(B)** Among these fatal cases, the three most frequently reported AEs were visual impairment, confusional state and headache. Bar colours indicate the corresponding primary SOC.

Table S1. Annual case counts of adverse event reports (AERs) associated with fluoroquinolone (FQ) treatment, based on data from the FDA Adverse Event Reporting System (FAERS) Public Dashboard, 2011 - 2024.

Table S2. Demographic characteristics of adverse event reports (AERs) associated with fluoroquinolone (FQ) treatment, based on FAERS data, 2011 - 2024.

Table S1. Annual case counts of adverse event reports (AERs) associated with fluoroquinolone (FQ) treatment, based on data from the FDA Adverse Event Reporting System (FAERS) Public Dashboard, 2011 - 2024.

	Case Counts				
**Year**	**Psychiatric**	**Eye-related**	**Both**	**Other AERs**	**Total**
2011	487	164	59	2,844	3,554
2012	358	144	84	1,886	2,472
2013	340	148	93	1,966	2,547
2014	429	149	142	1,990	2,710
2015	504	202	124	2,680	3,510
2016	664	234	191	2,956	4,045
2017	607	271	146	2,778	3,802
2018	643	246	200	3,425	4,514
2019	750	244	165	3,684	4,843
2020	504	234	109	2,390	3,237
2021	414	123	115	1,790	2,442
2022	316	144	52	1,334	1,846
2023	424	195	163	2,004	2,786
2024	346	137	90	2,014	2,587

Table S2. Demographic characteristics of adverse event reports (AERs) associated with fluoroquinolone (FQ) treatment, based on FAERS data, 2011 - 2024.

	**Ciprofloxacin (N = 12,970)**	**Levofloxacin (N = 10,636)**	**Moxifloxacin (N = 4,425)**	**Ofloxacin (N = 514)**	**Gemifloxacin (N = 82)**	**Delafloxacin (N = 72)**	**Combination (N = 16,196)**	**Total (N = 44,895)**	***P*-value**
Sex									1.23E-67
Male	4,863 (37.49%)	3,786 (35.60%)	1,522 (34.40%)	198 (38.52%)	21 (25.61%)	24 (33.33%)	6,308 (38.95%)	16,722 (37.25%)	
Female	7,011 (54.06%)	5,509 (51.80%)	2,354 (53.20%)	258 (50.19%)	57 (69.51%)	36 (50.00%)	8,793 (54.29%)	24,018 (53.50%)	
Not Specified	1,096 (8.45%)	1,341 (12.61%)	549 (12.41%)	58 (11.28%)	4 (4.88%)	12 (16.67%)	1,095 (6.76%)	4,155 (9.25%)	
Age									6.61E-138
Median (Min - Max)	53 (37 - 68)	57 (44 - 70)	58 (43 - 72)	49 (31 - 65.5)	38 (29 - 46)	66.5 (47.5 - 74.5)	59 (44 - 71)	57 (42 - 70)	
Age Group									*P* < 0.0001
0 - 17	225 (1.73%)	143 (1.34%)	49 (1.11%)	37 (7.20%)	2 (2.44%)	1 (1.39%)	327 (2.02%)	784 (1.75%)	
18 - 34	1,749 (13.48%)	785 (7.38%)	332 (7.50%)	62 (12.06%)	22 (26.83%)	4 (5.56%)	1,540 (9.51%)	4,494 (10.01%)	
35 - 64	4,665 (35.97%)	3,984 (37.46%)	1,345 (30.40%)	139 (27.04%)	35 (42.68%)	8 (11.11%)	6,359 (39.26%)	16,535 (36.83%)	
65+	2,879 (22.20%)	2,588 (24.33%)	1,024 (23.14%)	89 (17.32%)	3 (3.66%)	13 (18.06%)	5,363 (33.11%)	11,959 (26.64%)	
Not Specified	3,452 (26.62%)	3,136 (29.48%)	1,675 (37.85%)	187 (36.38%)	20 (24.39%)	46 (63.89%)	2,607 (16.10%)	11,123 (24.78%)	
Reported Country or Continent									*P* < 0.0001
United States	5,037 (38.84%)	5,142 (48.35%)	2,176 (49.18%)	212 (41.25%)	6 (7.32%)	63 (87.50%)	6,260 (38.65%)	18,896 (42.09%)	
Great Britain	1,674 (12.91%)	219 (2.06%)	23 (0.52%)	40 (7.78%)	0 (0.00%)	0 (0.00%)	2,428 (14.99%)	4,384 (9.77%)	
Europe	3,110 (23.98%)	2,474 (23.26%)	677 (15.30%)	163 (31.71%)	36 (43.90%)	2 (2.78%)	3,432 (21.19%)	9,894 (22.04%)	
Asia	304 (2.34%)	320 (3.01%)	835 (18.87%)	31 (6.03%)	3 (3.66%)	0 (0.00%)	761 (4.70%)	2,254 (5.02%)	
North America	518 (3.99%)	145 (1.36%)	148 (3.34%)	1 (0.19%)	1 (1.22%)	0 (0.00%)	604 (3.73%)	1,417 (3.16%)	
South America	121 (0.93%)	41 (0.39%)	75 (1.69%)	0 (0.00%)	12 (14.63%)	7 (9.72%)	93 (0.57%)	349 (0.78%)	
Africa	84 (0.65%)	20 (0.19%)	16 (0.36%)	4 (0.78%)	2 (2.44%)	0 (0.00%)	57 (0.35%)	183 (0.41%)	
Middle East	41 (0.32%)	8 (0.08%)	26 (0.59%)	3 (0.58%)	1 (1.22%)	0 (0.00%)	53 (0.33%)	132 (0.29%)	
Australia or New Zealand	66 (0.51%)	1 (0.01%)	5 (0.11%)	0 (0.00%)	0 (0.00%)	0 (0.00%)	39 (0.24%)	111 (0.25%)	
Not Specified	2,015 (15.54%)	2,266 (21.31%)	444 (10.03%)	60 (11.67%)	21 (25.61%)	0 (0.00%)	2,469 (15.24%)	7,275 (16.20%)	
Reporter Type									1.28E-113
Consumer	6,791 (52.36%)	4,960 (46.63%)	1,674 (37.83%)	238 (46.30%)	50 (60.98%)	22 (30.56%)	8,585 (53.01%)	22,320 (49.72%)	
Healthcare Professional	5,802 (44.73%)	5,279 (49.63%)	2,668 (60.29%)	241 (46.89%)	26 (31.71%)	47 (65.28%)	7,329 (45.25%)	21,392 (47.65%)	
Not Specified	377 (2.91%)	397 (3.73%)	83 (1.88%)	35 (6.81%)	6 (7.32%)	3 (4.17%)	282 (1.74%)	1,183 (2.64%)	
Outcomes									*P* < 0.0001
Died	286 (2.21%)	286 (2.69%)	89 (2.01%)	14 (2.72%)	0 (0.00%)	4 (5.56%)	484 (2.99%)	1,163 (2.59%)	
Life-threatening	542 (4.18%)	464 (4.36%)	343 (7.75%)	16 (3.11%)	5 (6.10%)	1 (1.39%)	874 (5.40%)	2,245 (5.00%)	
Hospitalised	1,941 (14.97%)	1,938 (18.22%)	586 (13.24%)	65 (12.65%)	17 (20.73%)	3 (4.17%)	3,037 (18.75%)	7,587 (16.90%)	
Disabled	2,259 (17.42%)	1,611 (15.15%)	225 (5.08%)	45 (8.75%)	7 (8.54%)	0 (0.00%)	3,663 (22.62%)	7,810 (17.40%)	
Other Serious	4,523 (34.87%)	4,178 (39.28%)	1,778 (40.18%)	174 (33.85%)	30 (36.59%)	18 (25.00%)	5,916 (36.53%)	16,617 (37.01%)	
Non-serious	3,419 (26.36%)	2,159 (20.30%)	1,404 (31.73%)	200 (38.91%)	23 (28.05%)	46 (63.89%)	2,222 (13.72%)	9,473 (21.10%)	
